# Ion-Size Controlled Non-Classical Crystallization of Metal-Oxide Nanoparticles Covered with a Few Highly Charged Ligands

**DOI:** 10.1021/jacs.5c18213

**Published:** 2026-01-21

**Authors:** Mark Baranov, Jintumol Mathew, Aranya Kar, Nitai Leffler, Arti Joshi, Shubasis Roy, Gal Gan-Or, Vladimir Ezersky, Petr Král, Ira A. Weinstock

**Affiliations:** † Department of Chemistry, 26732 Ben-Gurion University of the Negev, Beer Sheva 84105, Israel; ‡ Ilse Katz Institute for Nanotechnology Science, 26732 Ben-Gurion University of the Negev, Beer-Sheva 84105, Israel; § Department of Chemistry, University of Illinois Chicago, Chicago, Illinois 60607, United States; ∥ Department of Physics, Pharmaceutical Sciences, and Chemical Engineering, University of Illinois Chicago, Chicago, Illinois 60607, United States

## Abstract

Densely ligated metal and metal-oxide nanoparticles (NPs) tend to assemble into superlattices (SLs) of different symmetries determined by a delicate balance of dominant interparticle forces. However, the organic protecting ligands typically used to stabilize NPs often block substrate access to their reactive surfaces, acting as an insulating barrier that prevents electronic coupling and limits optoelectronic activities. We now report that the addition of K^+^ cations to aqueous solutions of 2 nm metal-oxide nanocrystals (NCs) with exposed surfaces due to complexation on average by eight polyoxometalate (POM) ligands promotes their reversible assembly into soluble SLs. Time-resolved cryo-TEM revealed the initial formation of fractal aggregates whose branching nodes serve as nuclei for the nonclassical self-limiting crystallization of dynamic, negatively charged, and uniformly sized 110 ± 20 nm body-centered cubic (BCC) crystals. Atomistic molecular dynamics simulations revealed that K^+^ cations promote dynamical association of 8 POMs ligated to different NCs, causing their assembly into crystals, whereas small Li^+^ ions randomly but transiently bind to the POM ligands, thereby dynamically changing the effective symmetries of individual NCs, preventing their crystallization. Unlike when organic protecting ligands are used, the exposed metal-oxide surfaces of the small-ion BCC SLs (K^+^ form) are stabilized by redox- and photochemically active POM-anion ligands. The findings thus introduce an attractive approach to the rational design of functional small-ion metal-oxide NC SLs.

## Graphical abstract

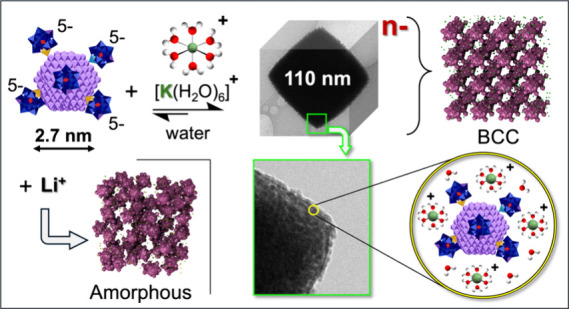

## Introduction

In recent decades, a wide variety of ligand-stabilized NPs have been prepared and crystallized into many different superlattices (SLs),^1–7^ and even quasicrystals.^8^ Through combined experimental and modeling studies,^9^ it has been revealed^5^ how the NP type and morphology,^10^ ligand type and size,^2^ NP concentration, solvent type, and temperature^11–13^ can affect the crystalline phase^12^ and polymorphism.^11,14^ In addition, binary and small-ion SLs are also formed, respectively, when highly charged NPs are neutralized by oppositely charged NPs of different types or by the addition of small ions.^15–17^

Metal-oxide nanocrystal (NC) assembly can give rise to unique electronic,^18^ electrochemiluminescent,^19^ magnetic,^20^ electrochromic^21^ and plasmonic^22,23^ properties. However, the organic protecting ligands typically used to stabilize metal-oxide NCs^24^ often block access to their reactive surfaces, and act as insulating barriers,^25^ preventing electronic coupling^18^ and limiting optoelectronic activities.^20,21^ To prepare functional materials comprised of assembled NCs with chemically or electronically active surfaces,^1,18,26^ protecting-ligand exchange^18,25,27^ and oxidative stripping methods have been developed.^19,27,28^ Alternatively, NC surfaces could be covered by just a few ligands of high rigidity, provided that NCs of this type could be controllably assembled.

Recently, redox and photochemically active^29^ metal-oxide cluster-anions called polyoxometalates (POMs) have been coordinatively attached to metal-oxide NC-cores,^30,31^ giving macroanion-like complexes.^29,32–37^ Unlike organic ligands, bulky POMs bind directly to a limited fraction of atoms at the reactive surfaces of complexed NC cores, leaving them largely exposed to solution. The large negatively charged POM ligands might be partially organized on the NC-surfaces, with close to equidistant separations. These highly charged POM-NCs are soluble in water, where they can be neutralized by alkali-metal counter-cations.^38^ Until now, however, it was not known whether POM-complexed NCs could be crystallized.

We herein show that upon addition of K^+^ cations to aqueous solutions of 2 nm ε-MnO_2_ NCs, each sparingly coordinated by approximately eight negatively charged polyoxometalate ligands, the macroanion-like complexes (**1**) assemble into soluble, 110 ± 20 nm, body-centered cubic (BCC) SLs. The relatively small SL size, and modest rate of structural evolution allows for time-resolved cryo-TEM imaging of progress from individual particles to final crystalline structures, during which initially formed fractal-aggregate branching nodes serve as nonclassical nuclei. Atomistic molecular dynamics (MD) simulations revealed that hydrated and mobile [K­(H_2_O)_6_]^+^ ions stabilize electrostatic POM ligand–ligand binding of different complexes, **1**, into BCC lattices. Moreover, the residual negative charge that results from partial dissociation of the hydrated K^+^ ions leads to self-limiting assembly of uniformly sized small-ion SLs.

## Results

### Preparation of POM-Complexed NCs

POM-complexed ε-MnO_2_ NCs **1** (Figure 1a) were prepared by adding MnO_4_^–^ to a hot aqueous solution of [AlCr^III^W_11_O_39_]^6–^, and refluxing under air for 2 h (see Experimental section and Figure S1). After workup, the yield of **1** was 46.2%, based on Mn. Cryo-TEM images of these samples revealed individual particles with an average size of 2.7 ± 0.2 nm (Figure 1b and Figure S2).

**Figure 1:**
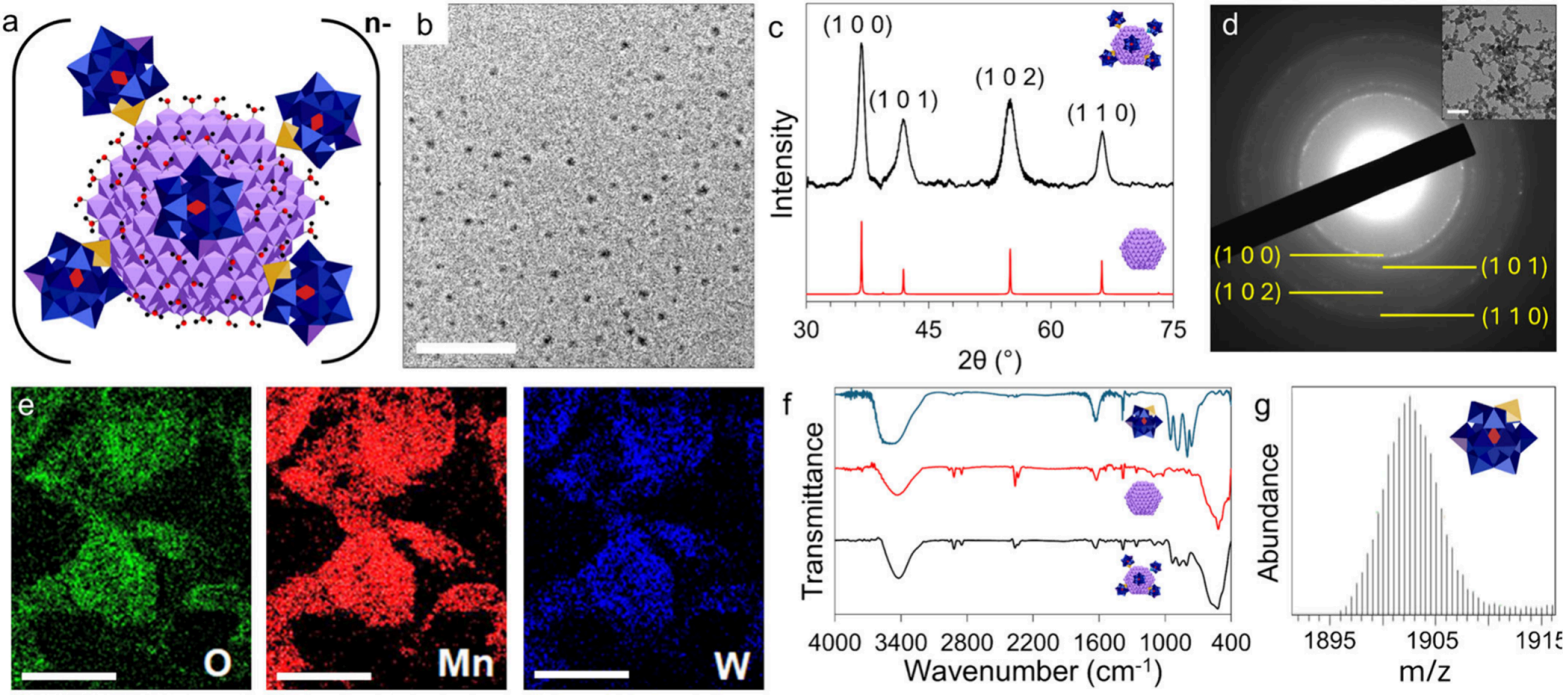
**Characterization of the core and ligands of 1.** (a) Model in polyhedral notation of POM ligands on an ε-MnO_2_ NC. Color code: purple octahedra represent MnO_6_ units, blue octahedra represent WO_6_ units, yellow octahedra represent CrO_6_ units, red tetrahedra represent AlO_4_ units, black spheres represent hydrogen atoms, and red spheres represent oxygen atoms. (b) Cryo-TEM image of a vitrified solution of **1** after 5 days of dialysis, showing individual NCs (scale bar 20 nm). (c) Powder X-ray diffraction of **1** (black curve), showing the characteristic peaks for ε-MnO_2_ (red curve). (d) Selected area electron diffraction showing a ring pattern corresponding to the hexagonal phase of ε-MnO_2_. Inset: TEM image of dried **1** from which the SAED was taken (scale bar 100 nm). (e) EDS mapping showing a homogeneous distribution of Mn, O, and W (scale bars 500 nm). (f) The FT-IR spectrum of **1** (black curve) shows the characteristic peaks associated with both [AlCr^V^W_11_O_40_]^6–^ (blue curve, TBA salt) and MnO_2_ (red curve). (g) ESI-MS spectrum of the POM ligands (z = 2^+^) liberated from the NC surface by ascorbic-acid reduction of the ε-MnO_2_ cores.

Powder X-ray diffraction (XRD), electron diffraction (Figures 1c and 1d, respectively) and high- resolution TEM (Figure S3) identified that **1** had ε-MnO_2_ cores.^39,40^ Energy-dispersive X-ray spectroscopy (EDS) and elemental mapping confirmed the presence of Mn, O, and W atoms (Figure 1e and Figure S4). The ligating POM ligands were characterized by FTIR and electrospray ionization mass spectrometry (ESI-MS). The FT-IR spectrum of **1** (Figure 1f, black curve) in the 700–1000 cm^–1^ region displayed bands characteristic of independently prepared [AlCr^V^W_11_O_40_]^6–^ (tetra-*n*-butylammonium (TBA) salt; Figure 1f, blue curve),^41^ while the intense broad band at 400–700 cm^–1^ matches that of colloidal MnO_2_ (Figure 1f, red curve). For ESI-MS, ascorbic acid was used to reduce the ε-MnO_2_ cores of the K^+^ salt of **1**, and the liberated POM ligands were precipitated by addition of TBABr and dissolved in acetonitrile. ESI-MS then revealed z = +1 and +2 ions with envelopes centered at 3804 and 1902.5 *m*/*z*, respectively, closely matching the simulated masses of KHMn^II^TBA_4_[AlCr^V^W_11_O_40_]^2+^ and KMn^II^TBA_4_[AlCr^V^W_11_O_40_]^+^ (Figure 1g and Figure S5). The presence of Cr^V^ was confirmed by electron paramagnetic resonance (EPR) spectroscopy, in line with reports that Cr^V^ is not readily reduced by ascorbate^42,43^ (Figure S6).

The number of POM ligands complexed to each ε-MnO_2_ NC was estimated by data from inductively coupled plasma optical emission spectroscopy (ICP-OES). They provided an atom-percent ratio of W to Mn, corresponding to an average of 7.7 [AlCr^V^W_11_O_40_]^6–^ ligands per 2.7 nm NC, each containing 360 Mn atoms (see structural models in Figures 1a and 1e, and Table S1). Based on a footprint of 1 nm^2^ for the 0.56 nm radius Keggin anion, ca. 20 POM ligands would be required to provide monolayer coverage of the NC surface.

X-ray photoelectron spectroscopy (XPS) of **1** revealed the presence of Mn^III^ and Mn^IV^ (Figure S7),^44^ consistent with reports that ε-MnO_2_ NCs contain Mn^III^ at the particle surfaces.^45–47^ The hexagonal close packing of O atoms^48^ within the ε-MnO_2_ phase precludes the presence of Mn­(III) sites within the crystal. Moreover, the Mn^III^/Mn^IV^ ratio observed by XPS corresponds to the value expected if surface Mn ions of the 2.7 nm diameter cores of **1** are all in the Mn^III^ oxidation state. Further evidence for Mn^III^ was provided by cyclic voltammetry (Figure S8). The surface Mn^III^ ions of ε-MnO_2_ NCs were coordinated by surface hydroxyl groups.^45^ Deconvolution of the O 1s peak indicated the presence of metal–oxygen–metal linkages, adsorbed water, and metal–hydroxide groups, the latter associated with surface Mn^III^ sites. The abundance of surface hydroxide groups was consistent with the absence of K^+^ when analyzed by ICP-OES after 5 days of dialysis. The hydroxide surface was sufficiently basic to become protonated, resulting in a decrease in pH from 7 to 5 as Mn^III^–OH sites were converted to [Mn^III^–OH_2_]^+^.

Based on ∼7.7 [AlW_11_O_39_Cr^V^]^4–^–O^–^ donor ligands per NC, each assigned a charge of 5- by including half of the 2- charge of the shared μ-O donor atom, ∼38 cations were needed to balance the POM-NC charge. Full protonation of the POM-NC would correspond to ∼38 [Mn^III^–OH_2_]^+^ surface sites balancing the ca. 38- charge of the ligating POM polyanions. However, partial dissociation of protons from the [Mn^III^–OH_2_]^+^ sites gives rise to a zeta potential of ζ ≈ –60 mV (Figure S9). These negatively charged complexes, **1**, are highly soluble, as indicated by their optically transparent solutions.

### Nonclassical Self-Assembly of POM-NCs

Next, we examined whether **1** could self-assemble when incremental amounts of alkali-metal cations are added in their solutions. First, K^+^ was added to **1** solutions in the ratios of 8.8, 13.2, and 17.6 equiv per POM ligand, corresponding to K^+^ concentrations of 0.13 mM, 0.20 mM, and 0.27 mM, respectively. After the samples were equilibrated for 7 days at ambient temperature, cryo-TEM imaging showed a gradual formation of assemblies (Figure S10). At 8.8 equiv, only fractal NC-aggregates were observed. At 13.2 equiv, discrete amorphous assemblies were formed, but at 17.6 equiv, well-defined cubic lattices were obtained. Addition of excess K^+^ (10 mM, 650 eq. K^+^ to POM) resulted in clumping of the crystallites into larger aggregates (Figure S11).

Further details of the lattice formation (17.6 equiv of K^+^) were examined by cryo-TEM at specific time intervals (Figure 2, panels a–h). Within a few seconds of K^+^ addition, the minimum time needed for mixing and sampling, cation association had caused the negatively charged POM-NCs, **1** (Figure 2a), to assemble into the fractal aggregates shown in Figure 2b, comprised of cross-linked filaments (see additional images in Figure S12).

**Figure 2:**
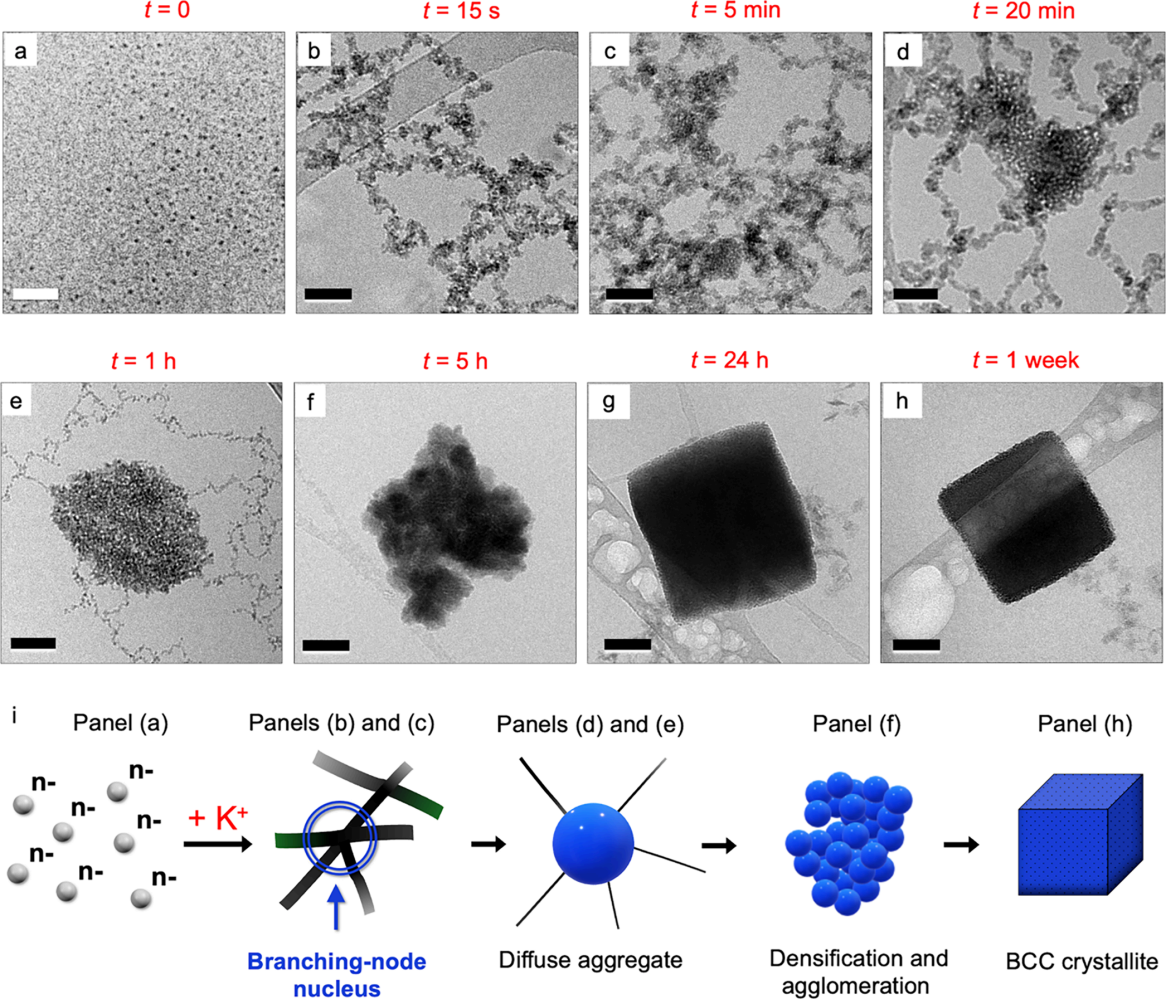
**Time-resolved cryo-TEM imaging of POM-NC lattice formation and the role of branching-node nuclei.** (a) Individual POM-complexed ε-MnO_2_ NCs (**1**) prior to K^+^ addition (scale bar = 20 nm) and (b-h) cryo-TEM images acquired after adding 17.6 equiv of K^+^ per POM ligand to the solution in (a) (all scale bars are 50 nm), revealing structures formed along the pathway from individual NCs to the small-ion lattice in (h). Within seconds of K^+^ addition, the negatively charged NCs form NC-aggregate strands (b), whose branching nodes serve as nonclassical nucleation sites (c, d) for the growth of amorphous 3D aggregates (e, *t* = 1 h). Over the next 4 h, these 3D aggregates densify and agglomerate to give dense, amorphous aggregates (f). By *t* = 24 h (g), morphological definition shows the result of a transition, based on internal ordering, toward a symmetry-defined phase that, with additional time leads to BCC lattices (h). Panels (b) to (h) share identical 50 nm scale bars to better visualize the relative sizes of the branching-node nuclei and subsequent structures along the pathway to symmetry-defined lattices. (i) Schematic illustration of the evolution from individual NCs (gray spheres) to a BCC crystallite (blue cube). Addition of K^+^ induces the formation of aggregate strands with multiple branching nodes (gray strands) which act as nonclassical nuclei that grow into diffuse aggregates which densify and agglomerate to give amorphous aggregates that organize into BCC lattices.

Fractal aggregates are commonly observed in solutions of charged colloidal NCs. Image analysis using a box-counting method^49^ yielded an average fractal parameter of *D* = 1.75 ± 0.04 (Supporting Information Figures S13–S15 and Table S2). Images in Figure 2c and d, obtained after 5 and 20 min, revealed that the branching nodes of these fractal aggregates serve as nonclassical nuclei for a continued growth into larger, yet still relatively diffuse clusters of 100–150 nm, shown in Figure 2e (60 min; see additional images in Figures S16–S21). This role of the branching nodes is more easily appreciated by noting that the scale bars in panels b to h in Figure 2 are identical (50 nm). This uniform scaling also reveals that the 40 min progression in panels 2d to 2e, from *t* = 20 min to 1 h, involves a significant decrease in the thickness and numbers of strands attached to the growing nodal aggregate, with the branching-node nucleus appearing to incorporate the filaments at whose intersection it originally formed.

Over the next 4 h (Figure 2e–f), a dramatic increase in contrast was observed, indicative of significant densification (Figure 2f, *t* = 5 h). Notably, the dense structure was similar in size to the branching node in Figure 2e (*t* = 1 h). This suggested that in addition to densification, multiple nodal structures had agglomerated to give the object in Figure 2f (see related *t* = 5 h images in Figure S19). Further densification overlapped in time with phase formation, represented by the pseudocubic structure in Figure 2g (*t* = 24 h; additional images in Figure S20 reveal various degrees of morphological definition). The process culminated in well-formed crystallites (Figure 2h and Figure S21).

Figure 2 revealed that branching nodes of fractal NC aggregates^50,51^ can serve as nonclassical crystallization nuclei.^52^ The energetics of branching-node nuclei formation and growth was investigated by isothermal titration calorimetry (ITC), and recorded throughout the instrumental limit of 4 h, during which 17.6 equiv of K^+^ per POM ligand were incrementally added to a solution of **1** (proton form). Heat was released after each K^+^ injection, (Figure S22a) indicating that the reaction was exothermic. Moreover, the consistent release of heat throughout titration by an excess of K^+^ was indicative of relatively weak interactions, with no evidence of saturation.

Fitting of integrated values of heat released to an independent binding site model^53^ (Figure S22b and Table S3) revealed a modest association constant of *K* = 3.7 mM, a large change in enthalpy of Δ*H* = −33.6 kcal**·**mol^–1^, and a large entropy contribution of −*T*Δ*S* = 30.2 kcal**·**mol^–1^ (*T* = 298 K) to the total Gibbs free energy of NC-binding of Δ*G* = Δ*H* – *T*Δ*S* = −3.4 kcal**·**mol^–1^. The large, negative Δ*H* value suggested that association between K^+^ and **1** was not accompanied by enthalpically unfavorable dehydration of [K­(H_2_O)_6_]^+^ ions.^54^ The large, positive −TΔ*S* value also did not support entropically favorable release of water molecules that would occur upon dehydration of K^+^ ions. These results provided evidence for a solvent-separated ion coupling between [K­(H_2_O)_6_]^+^ cations and **1** during nucleation and agglomeration (i.e., the first 4 h of assembly).

The delicate balance between ΔH and -TΔ*S* during branching-node formation provides a thermodynamic basis for discussing their role as nonclassical nuclei. During the first seconds of assembly, degrees of translational freedom of both **1** and [(H_2_O)_6_K]^+^ ions decrease as individual (0D) particles (Figure 2a) form branched pseudo-1D strands (Figure 2b). The strands retain significantly larger degrees of translational freedom than would compact 3D aggregates, even though the long strands are connected to one another via nodes. Consistent with the fine balance between ΔH and −TΔ*S* (Table S3), the nodes contain more cations and favorable electrostatic interactions, which attenuate the entropy decrease associated with their formation. As such, the branching nodes form via a mildly exergonic step that bypasses the large nucleation energy barriers associated with classical nucleation theory, including the high-surface-energy phase-defined nuclei typical of Ostwald ripening.^55^

Notably, fractal aggregates are typical of colloidal metal-oxides,^50,51^ and are observed in solutions of ionic prenucleation assemblies that serve as precursors to calcium-based crystalline inorganic materials.^52,56^ While available published data are insufficient to draw general conclusions, the present findings suggest that, where fractal aggregates are observed during early stages of NP assembly, their branching nodes might serve as nonclassical nuclei.

### Structural Analysis of POM-NC SLs

Small-angle X-ray scattering (SAXS) is a definitive method for establishing SL phase. Unfortunately, efforts to obtain informative SAXS data were not successful. There are several reasons for this. First, the tungsten ligands strongly absorb X-rays, resulting in very weak scattering intensities. Second, the tungstate clusters are unstable under prolonged exposure to the beam, leading to precipitation. Third, unlike larger insoluble SLs, which are routinely investigated by SAXS, the SLs reported here are dynamic solution-state structures. As a result, the precise positions of the NCs in the SLs fluctuate to a greater extent than do NCs in solid-state SLs. Therefore, a quantitative profiling method based on cryo-TEM analysis was used to identify the phase (see Methods in the Supporting Information), and the results are confirmed by atomistic molecular dynamics simulations discussed below. First, a tilted-grid cryo-TEM analysis of the finally formed SLs revealed a cubic morphology (Figure 3a), suggesting a centrosymmetric cubic space group. To refine this further, profile analysis of a typical SL (Figure 3b, left) based on 2.5 nm radius NCs, calculated by including the POM ligands, gave an average NC spacing of Δ*x* = 2.84 nm. This value was determined by minimizing the uncertainty in the experimental profile shown on the right in Figure 3b using a method described in the Supporting Information. For a BCC lattice comprised of 2.5 nm-radius spheres (left in Figure 3c) the calculated lattice constant of 5.8 nm yields a spacing of Δ*x* = 2.9 nm in the [100] projection (right in Figure 3c), closely matching the experimentally determined value. Notably, modeling of the BCC lattice with each NC represented by a sphere, with the center being the most electron dense, provided good correlation with the observed profile plot (right in Figure 3c). The close fit to BCC packing was confirmed by comparison with spacings calculated for FCC packing of 2.5 nm spheres, which yielded a value of Δ*x* = 3.53 nm. The calculated spacing profile for the FCC structure is shown at the top-right of Figure 3b. Critically, modeling other lattice types such as HCP, SC, and tetragonal gave Δ*x* values even larger than that for FCC, leaving BCC as the definitively best fit.

**Figure 3:**
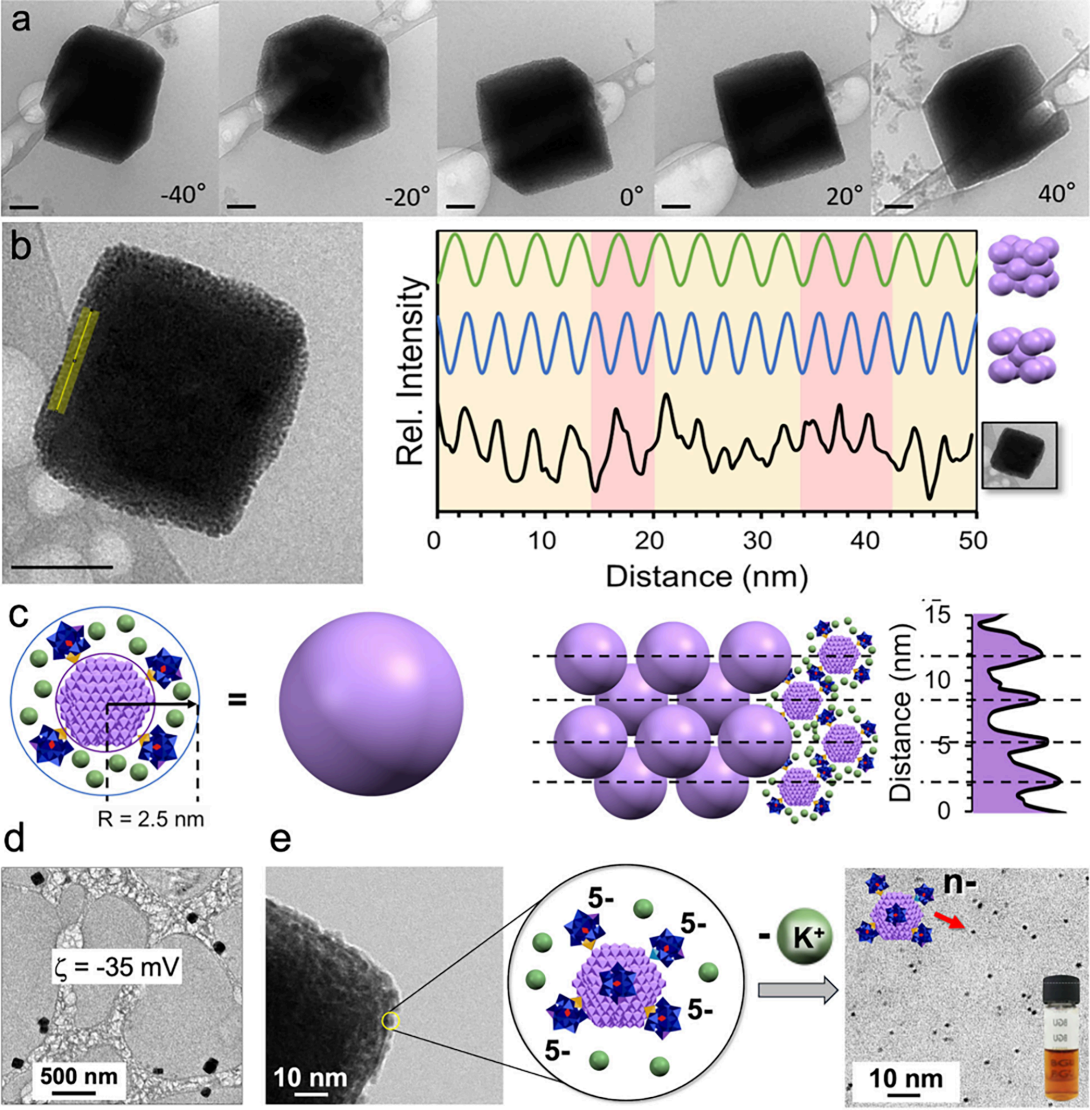
**Morphology, phase, and reversibility of POM-NC lattices.** (a) Tilting series confirming the cubic nature of the crystallites formed after adding 17.6 equiv of K^+^ to **1**, demonstrating that the three-dimensional assembly adopts a high-symmetry lattice. (b) Left: Cryo-TEM image of a lattice. The yellow line indicates where the profile plot was taken. Right: The black trace is the corresponding intensity profile, revealing periodic ordering of individual NCs. (Prior to structure determination, the uncertainty was minimized (see Methods in the Supporting Information). Simulated periodicities for body-centered cubic (BCC) and face-centered cubic (FCC) lattices (blue and green traces, respectively) show that even the unoptimized experimental data (highlighted by yellow regions) closely match a BCC arrangement, with occasional lattice irregularities indicated by red regions. (c) A model of the BCC lattice, with each nanocrystal represented as a purple sphere, accurately reproduces the observed intensity profile. Scale bars = 50 nm. (d) Low magnification cryo-TEM image showing several crystallites. (e) High magnification cryo-TEM image of a crystallite (left), and an illustration (center) of an individual NC with its hydrated [K­(H_2_O)_6_]^+^-ions that comprise a lattice building unit. Cryo-TEM image at right shows individual complexes, **1** (illustrated in the inset) after using dialysis to remove added K^+^ ions, resulting in complete exchange of K^+^ counter cations by protons.

Despite the close fitting of the profile analysis to BCC packing, the crystal was imperfect due to positional disorder (thermal fluctuations, finite correlation lengths), dynamic motion of particles and ligands, ligand shells of finite thickness and possible inhomogeneity, and size and shape polydispersity of the NCs. As a result, peaks in the experimental line profile (right in Figure 3b) are broadened, and a few maxima are missing, leading to occasional larger gaps between peaks. These deviations from the ideal pattern are not unexpected in a dynamic, solvated SL.

The crystallites formed had uniform edge lengths of 110 ± 20 nm (Figure S23). To understand this better, note that the static phase-defined particles frozen in vitreous water matrices during cryo-TEM sample preparation (Figure 3b) were negatively charged. During assembly at pH = 5.5, zeta potentials changed from ca. ζ = −55 mV for individual NCs (Figure S7) to ζ = −35 mV for fully formed crystals (Figure 3d and Figure S24). The negative charge of the formed lattices, despite an excess of added K^+^, reflects entropically driven retention of hydrated cations in bulk solution. While the negative charge of the crystallite contributes to its solubility, the residual charge density within the lattice, caused by incomplete K^+^ shielding of **1**, resulted in their self-limiting terminal growth^57–60^ and size uniformity (see the Discussion section for more details). Consistent with the equilibrium thermodynamics associated with self-limiting assembly, lattice formation was reversible, as demonstrated by using dialysis to remove labile K^+^ ions, after which cryo-TEM images revealed individual complexes, **1** (Figure 3e).

Recently, lattices of charged NPs were stabilized by small multivalent ions.^16,61^ In this context, the ability of K^+^ ions to drive lattice formation (Figure 3e) prompted a closer look at other cations. We tested the assembly of **1** (2 μM solutions) in the presence of Li^+^, Na^+^, K^+^, Rb^+^, and Cs^+^, each at final concentrations of 5 mM. The sizes of amorphous aggregates, indicated by number-weighted hydrodynamic diameters (*D*_*h*_), from dynamic light scattering (DLS), increased from *D*_*h*_ = 48 ± 8 nm for Li^+^ to *D*_*h*_ = 229 ± 57 nm for Cs^+^ (Figure S25). This trend is consistent with solvent-separated ion-pairing with tungstate-based POMs,^38^ wherein association constants increase with the crystallographic sizes of alkali-metal cations. This is because the larger monovalent cations possess smaller hydrated radii, which decrease in the order: Li^+^ (3.40 Å) > Na^+^ (2.76 Å) > K^+^ (2.32 Å) > Rb^+^ (2.28 Å) > Cs^+^ (2.26 Å).^62^ The aqua cations with smaller hydrated radii more closely approach negatively charged POMs, resulting in greater decreases in Coulombic potential energy. As such, the increase in assembly size from Li^+^ to Cs^+^ (Figure S25) was initially viewed as evidence for solvent-separated ion-pairing.^63^

### Modeling the Self-Assembly of POM-NCs

To shed more light on these observations, we modeled the POM-NC systems by atomistic molecular dynamics simulations with realistic force-fields.^64^ First, we prepared an atomistic model of **1**, formed by an ε-MnO_2_ core of ∼2.4 nm diameter, H^+^ and OH^–^ groups randomly coordinated to half of the oxygen atoms and Mn­(III) ions exposed at the ε-MnO_2_ NC surface, respectively,^58^ and 8 randomly attached POM ligands. The atomic (ESP) charges of the model [AlW_11_O_40_Cr]^5–^ ligand were calculated in implicit water by DFT^65–69^ at the B3PW91/LANL2DZ level. The remaining force-field parameters of the ligand were taken from the literature.^70,71^ These 8 POM anions, [AlW_11_O_40_Cr]^5–^, were attached to the ε-MnO_2_ NC via μ_2_-O linkages formed between [AlW_11_O_39_Cr]^4–^-O^–^ ligands and the surface Mn ions.

The NC core was approximated more since it was less involved in the observed ligand–ligand coupling. In the bulk ε-MnO_2_ structure, +1 and −0.5 charges were assigned to Mn and O atoms, respectively. The NC surface was modified by attaching 85 OH^–^ and 84 H^+^ groups to Mn^3+^ and oxygen atoms, respectively. In these groups, the H and O atomic charges were approximated by charges present in water molecules. To figure the overall charging of **1**, we have assumed that before POM ligands are attached to the ε-MnO_2_ NC, its surface was neutral, reflecting its isoelectric point (pH = 5.5).

Each of the 8 POM ligands carries a nominal charge of 5e, which would give each POM-NC a total charge of 40e. However, due to a low local pH induced by each POM present close to the NC surface, this excess charge was expected to be compensated by a local protonation of the NC surface in the regions proximal to each POM. These compensating charges (protons) of ca. +4 were added on the surface Mn and O atoms within 4 Å from each POM, effectively reducing its charge to ∼1e, but forming with the charged POM a large dipole. Finally, a charge was added to the core of each NC to set its total charge q = 7e, to match the experimental zeta-potentials.

First, we performed separate simulations of one or two POM ligands in K^+^ or Li^+^ solutions, presented in Figure 4a. The simulated POM pairs are shown in the inset, where small Li^+^ cations are seen to strongly couple to POM terminal oxygens, each potentially losing 1–2 waters in their hydration shells.^72^ In contrast, larger K^+^ cations do not stick to POM ligands, but they shield them at close distances. This behavior is further evident from the radial pair distribution (RDF)^73^ functions, g­(r) (see Methods), of cations around the one or two POM ligands. The Li^+^ RDF revealed one large peak located at ∼6.65 Å distance from the POM center, which originates from Li^+^ directly bound to one of the POM terminal O^–^ (Figure 4a). In contrast, the K^+^ RDF (magnified by 2) revealed two small peaks located at ∼7.35 Å and ∼8.45 Å distances from the POM center. The first peak originates from K^+^ directly but weakly binding to POM terminal oxygens, while the second peak represents the same when water from the first hydration shell of K^+^ enters between these two charged binders. However, both peaks sit on a wide plateau, which contains much larger probability of K^+^ shielding POM from distance. There is also a slightly higher presence (RDFs) of both cations around the POM in the **1** systems.

**Figure 4:**
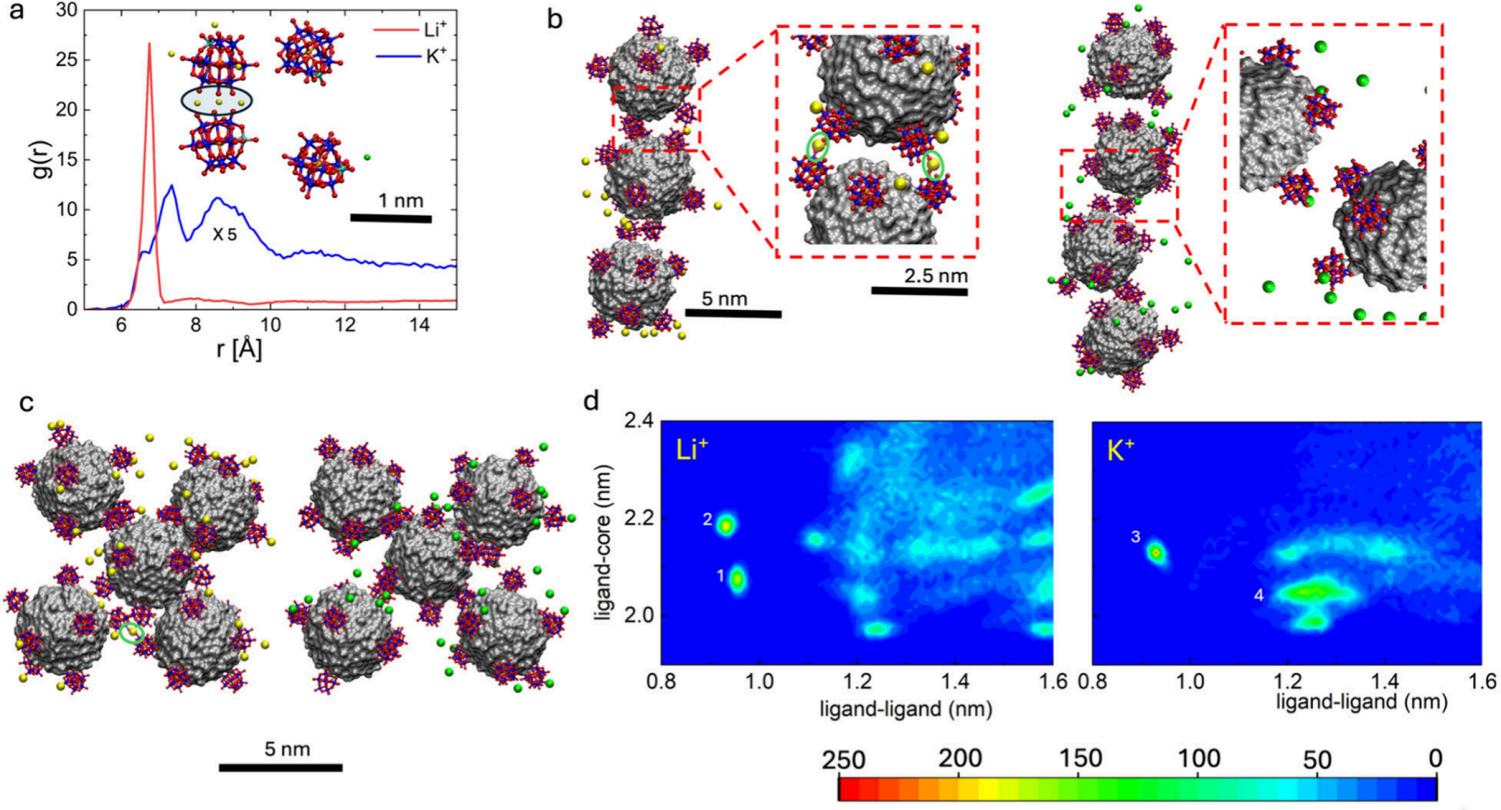
**Molecular dynamics simulations of POM-ligated NCs in Li** ^**+**^ **or K** ^**+**^ **-rich solutions.** (a) RDFs of Li^+^ or K^+^ cations around [AlW_11_O_40_Cr]^5–^, plotted as a function of the Al–Li^+^/Al–K^+^ distance in single POM and double POM systems (50 ns simulations; NpT ensemble, *T* = 300 K, *P* = 1 bar – conditions used everywhere). Inset: Snapshots of simulated POM pairs with Li^+^ or K^+^ cations (shown within 9 Å of Al; 50 ns, 27 nm^3^ water box, 10 K^+^ or 10 Li^+^). Blue and red balls indicate W and O atoms, respectively; yellow and green balls indicate Li^+^ and K^+^ ions, respectively. Water molecules are omitted for clarity. (b) Detail from Figure S26: Snapshots of POM-NC chains formed in the presence of Li^+^ (left) and K^+^ (right) cations, when 32 NCs are initially at center-to-center distances of 10 nm (100 ns, 15.6 × 10^3^ nm^3^ water box, 224 K^+^ or Li^+^). POM-NC cores are presented in gray. Insets: Magnified views of the attachment regions. (c) Snapshots of NCs in BCC crystal lattice arrangement in the presence of Li^+^ (left) and K^+^ (right) cations, where 64 NCs are initially placed at lattice constant a = 5.2 nm (100 ns, 6 × 10^3^ nm^3^ water box, 448 K^+^ or Li^+^). (d) Density plots obtained from (b) showing the correlations between nearby POMs on different POM-NCs and the surface distances of these POM-NCs (POM of NC1 and core center of NC2; NC-surface is positioned at ∼1.3 nm from the respective core center). Total weights under several dominant peaks are evaluated. In Li^+^, 1,942 and 1,965 weights correspond to peaks 1 and 2, respectively, while in K^+^, a 1,935 weight corresponds to peak 3, but a dramatic 14,530 weight corresponds to peak 4.

Next, we simulated freely assembling loose POM-NCs (Figure 4b) and a BCC crystal of POM-NCs with a lattice constant of a = 5.2 nm (Figure 4c). Bulk van der Waals (vdW) coupling between NC cores was added in the force field and implemented during these simulations^74^ (Methods). In Figure 4b and Figure S26 a,b, we can see that loose POM-NCs start to bind and form short irregular chains in the presence of either Li^+^ or K^+^ ions (100 ns). Typically, chains rather than clusters are observed to form when the overall charge of each NC is not fully compensated, leading to an overall Coulombic repulsion of different NCs (slightly compensated by their attraction due to their bulk vdW coupling).^75^ When Li^+^ ions are used, they randomly attach to terminal POM oxygens, eventually bridging over different NCs, and allowing the formation of NC chains. The transient but random Li^+^-POM-NC attachment hinders crystallization of these permanently changing NCs (Figures S27–S28).

In contrast, K^+^ ions do not directly attach to POM-NCs, but they help to stabilize a local dipole–dipole attractive coupling between neighboring NCs, where each dipole is formed by one POM ligand and its locally charged NC-surface. By freely moving around opposing dipoles formed by POMs on different NCs, K^+^ ions can stabilize their loose pairing, thereby providing suitable conditions for NC-crystallization in the BCC lattice type (each NC has ∼8 POMs).

In Figure 4c, POM-NCs crystals were initially prepared in the presence of either type of ions to test the ion dynamics in these lattices. The systems consisted of four layers of POM-NCs, with each layer comprised of 16 NCs arranged in a BCC lattice. To maintain positional stability during assembly, the NCs in the bottom layer were confined within a weak potential trap. While this constraint effectively limited the NCs translation, the particles significantly fluctuated. When these systems were simulated, the two types of ions had very different dynamics. The Li^+^ ions were attached to NCs and became practically immobile. In contrast, K^+^ ions were mostly free and capable of stabilizing the initial BCC POM-NC arrangement.

Figure 4d reveals more details about the NC-NC coupling within the systems from Figure 4b. Using the simulation trajectories, we correlate the (l-c) distance of a ligand center (NC1) and a core (NC2) with the (l-l) distance of a ligand center (NC1) and a ligand center (NC2). In the Li^+^ case, there are two regions around l-l ∼1.0 nm and l-c ∼2.1 nm, revealing that ligands stick via a Li^+^-cation bridge. The rest of this panel reveals random arrangement of NCs, preventing their crystallization. On the other hand, K^+^ mediates a loose ligand–ligand interaction around l-l ∼1.25 nm and l-c ∼2.05 nm (high probability density region), but much less ion-bridging. In this case, POM ligands remain closely attached but more widely separated, providing the necessary conditions for POM-NC crystallization.

### Electronic Coupling between Assembled NCs

Incremental increase in the concentration of K^+^ added to solutions of pure **1** resulted in correspondingly larger redshifts in the visible-light region (Figure 5a). When normalized relative to a solution of pure **1** (Figure 5b), the change in absorbance was most pronounced near 450 nm and was assigned to d-d transitions within the MnO_2_ cores. Over the concentration range studied (0 to 100 mM K^+^), the bandgap was red-shifted from 2.74 to 2.46 eV (Figure 5c), a change of ca. 6 kcal/mol.

**Figure 5:**
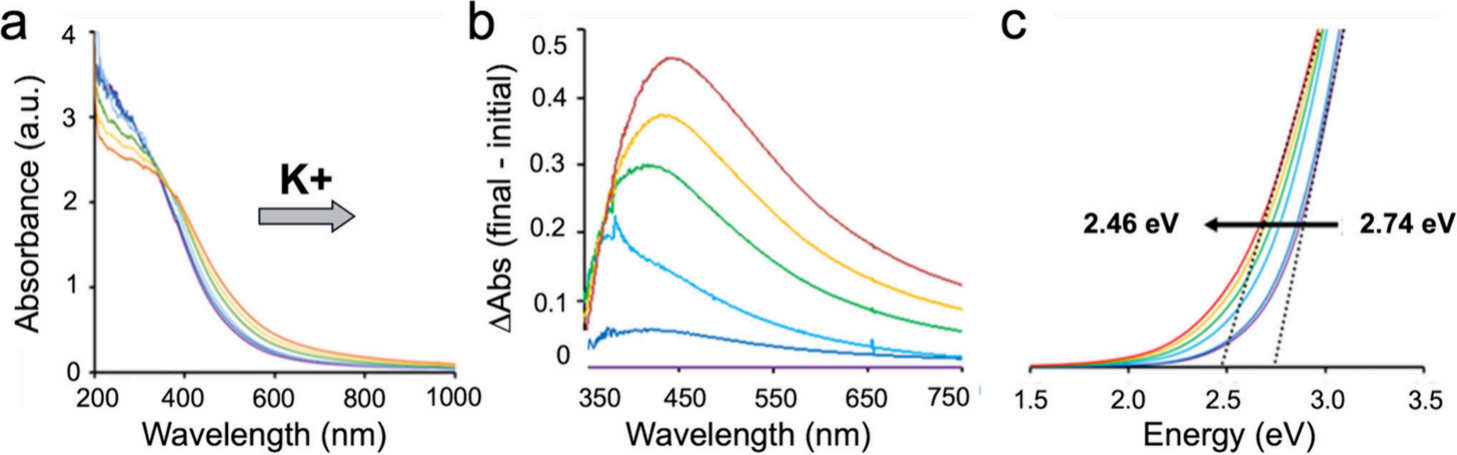
Assembly-induced changes in UV–visible absorption spectra. (a) Redshift in the UV–visible spectrum of solutions of **1** after incrementally larger additions of K^+^, from 0 to 100 mM. (b) Spectra from panel a) normalized by subtracting the absorbance spectrum of pure solutions of **1**. (c) Tauc plots showing that the redshift in the UV–vis spectra in (a) results in a bandgap change from 2.74 to 2.46 eV. Color coding of the curves refers to concentrations of K^+^; purple: 0 mM, dark blue: 5 mM, light blue: 10 mM, green: 25 mM, yellow: 50 mM, red: 100 mM.

While a redshift is not detected upon assembly of the 110 nm SLs of **1**, at 10 mM K^+^ (light-blue curves in Figure 5a–c), the SLs remain at approximately the same size but agglomerate into intact-SL aggregates (Figure S11; the basis for this behavior is discussed below). The formation of these extended structures correlates with greater electronic coupling between the entirely inorganic NC complexes. This phenomenon is a topic of ongoing research aimed at understanding the effects of mono-, di-, and trivalent cations on assembly size and shape, as well as on resultant degrees of aggregation-induced coupling of their d-d transitions.

## Discussion

### Comparison with Previously Reported SLs

Comments provided here locate SLs of **1** within the broader context of SL assembly and structure. While key developments are highlighted, the actual situation, which is more diverse and nuanced, has been comprehensively reviewed by Talapin.^4^

Early studies of micron-sized colloidal particles treated as hard spheres revealed that their crystallization to close-packed HCP and FCC phases is entropically driven.^76^ This was understood by comparing ordered phases with amorphous aggregates. Although the latter are less ordered, therefore suggesting less entropic cost in their formation, the translational motion of the particles is “jammed” by disorder. Although crystallization to close-packed phases leaves less void space, the increased order provides for greater translational freedom and a smaller decrease in entropy of formation. For differently sized NPs of the same type, a similar role of entropy also leads to close-packed SLs.^77^

For binary nanoparticle SLs (BNSLs), comprised of NPs with different compositions and protected by neutral organic ligands, an additional variable was found by Talapin^2^ to determine phase, namely, the “softness” (λ) of the NPs, defined as the ratio of ligand length to core radius, L/R. It was found that the ligand layer’s deformability led to changes in effective NC radii in response to specific coordination environments, in many cases resulting in packing arrangements less dense than those predicted by hard-sphere models.^78^ In those cases, the otherwise controlling role of entropy on phase was attenuated by van der Waals interactions and dispersion forces involving the particles and their ligand shells.

Electrostatically driven crystallization began with the use of protecting ligands with charged end groups, which raised the possibility of designing ionic-lattice BNSLs analogous to those formed by inorganic salts. Initially, the mixing of oppositely charged colloidal particles led to flocculation into amorphous aggregates. It was discovered that, because the screening layers at the nanoscale are similar in size to the particles themselves, smaller charged NPs were needed to induce crystallization.^79^ It was argued that the smaller NPs assume a role similar to that of ions^7,80,81^ in what was referred to as “a nanoscopic counterpart of Debye screening”.^80^ Notably, packing densities were lower than in single-phase FCC, ruling out entropy as the main driving force for crystallization.

The critical role of smaller charged NPs in crystallization of BNSLs was taken a step further by replacing the small particles with multiply charged molecular ions.^16^ For example, Au(0) NPs protected by thiolate ligands fitted with trimethylammonium (TMA^+^) end groups were found to crystallize into FCC lattices upon addition of EDTA^4–^, P_2_O_7_^4–^ or P_3_O_10_^5–^. However, as with many BNSLs formed from oppositely charged NPs, entropy no longer played a dominant role. Rather, small anion screening of the isotropically distributed charge of the densely packed protecting ligands enabled the Au NPs to assemble into close-packed structures. Notably, screening-anion charges of −3 or larger were required for crystallization, whereas even in excess, −2 anions such as SO_4_^2–^ and HPO_4_^2–^ did not induce aggregation.

By contrast, crystallization of **1** occurs upon the addition of monovalent K^+^ ions. The NC cores of **1** are relatively small, with diameters of 2.7 nm, and the 31- charge of their average number of 7.7 POM ligands is mostly balanced by protonation of the MnO_2_ surface. As such, each ligand possesses an average charge of −1. The ca. 8 POM ligands, each 1.12 nm in diameter, are not only close in size to the 1.35 nm radius of the complexed NC cores, but unlike monolayers of organic protecting ligand, are anisotropically distributed. According to MD simulations, these factors, combined with the much smaller volume occupied by the hydrated K^+^ cations relative to the NC core and its POM ligands, rule out the close packing required to achieve FCC or HCP phases. This result is similar to those obtained when sufficiently small charged NPs are used to assemble ionic BNSLs. For example, when charged colloidal particles are combined with much smaller spheres, the larger particles form a BCC lattice with CsCl structure.^79^ Six small spheres, analogous to the hydrated K^+^ ions in the present work, surround each of the phase-defining larger particles.

### The Energetic Basis for Self-Limiting Assembly

Quantitative information concerning the energetic basis for the self-limiting growth of similarly sized 110 nm SLs was obtained by considering the Gibbs free energy of K^+^ binding to POM ligands, determined by ITC, along with zeta potential values of individual complexes, **1**, and of finally formed SLs, and distances of hydrated K^+^ cations from POM ligands determined by MD simulations (see Methods in the Supporting Information).

Prior to K^+^ addition, the zeta potential (ζ) of **1** is −55 mV, which corresponds to an effective charge of – 7.5e. At the concentration of K^+^ added to drive SL assembly, the Debye length of each complex is λ_*D*_ = 17 nm. Before assembly, the NCs are ca. 98 nm apart, so a K^+^ ion in bulk solution experiences a near-zero potential. Based on the MD simulation, the binding distance of K^+^ was taken into account. Thus, when a hydrated K^+^ ion approaches **1** and binds to a ligating POM, it experiences a potential drop of either −0.179 or −0.155 V, based on the two POM-K^+^ distances determined by MD simulations. These voltages correspond to calculated *ΔG*_*calc*_ values of −4.1 and −3.6 kcal/mol, respectively, in good agreement with the experimental association energy, *ΔG* = −3.4 kcal/mol, obtained by ITC.

As assembly of **1** proceeds, K^+^ screening of the negative-charge accumulation reduces the zeta potential from ζ = −55 for individual particles, to −35 mV for the finally formed SL, assigning to the latter a total charge of −130e. This value, when distributed over all NCs on the SL surface, assigns an effective charge of only −0.036e per individual NC. At the POM-K^+^ distances of 7.45 and 8.45 Å determined by MD simulation, this value of −0.036e per NC gives potential drops for [K­(H_2_O)_6_]^+^ association of −0.861 and −0.744 mV, respectively, corresponding to *ΔG* values ca. 200 times smaller than for association with single, freely diffusing NCs. As such, the free energy change of bringing an additional K^+^ ion from bulk solution into association with the SL surface, as required to add an additional negatively charged NC, is insignificantly small. This results in an equilibrium aggregation number of approximately 28,000 NCs per SL, conceptually analogous to the thermodynamically controlled number of amphiphilic molecules in individual micelles at the critical micelle concentration.

At a much larger, 10 mM K^+^ concentration, the Debye length of each SL decreases to λ_*D*_ = 3 nm. The field around the SLs becomes highly localized, and K^+^ screening is essentially complete at nanometer distances. Under these conditions, residual attractive forces and ion-bridging effects dominate over long-range repulsion, leading to SL agglomeration, consistent with cryo-TEM observations (Figure S11). This behavior is fully consistent with the electrostatic framework described above.

## Conclusions

Upon the addition of K^+^ cations, metal-oxide NCs complexed by ∼8 highly charged POM ligands can reversibly assemble into soluble BCC SLs. In contrast, small Li^+^ ions randomly but transiently bind to the POM ligands, thereby dynamically changing the effective symmetries of individual NCs, preventing their crystallization. For K^+^-driven assembly, time-resolved cryo-TEM images not only document the early formation of commonly observed fractal aggregates but also reveal the role of structurally inherent branching nodes as nonclassical nuclei. Data from isothermal titration calorimetry (ITC) and atomic MD simulations show that the K^+^ cations remain hydrated and mobile throughout the assembly process, eventually forming dynamic solvent-separated POM pairs within the water-occupied interiors of the BCC SLs. As the SLs form, the negative charge density at their surfaces becomes too small to bind additional hydrated K^+^ ions, at which point further growth is no longer thermodynamically favorable, leading to a self-limiting stabilization of SL size.

In summary, we show that metal-oxide NCs whose reactive surfaces remain highly exposed due to stabilization by small numbers of rigid, yet redox- and photochemically active, POM-anion ligands can self-assemble into dynamic, uniformly sized crystals. As such, the findings introduce an attractive approach to the rational design of surface-exposed metal-oxide NC lattices.

## Experimental Section

### Synthesis of POM-Complexed ε-MnO_2_ Cores (1)

Synthesis of **1** (Figure S1) involves the addition of K_9_[AlW_11_O_39_]·13H_2_O (0.6518 g, 0.2 mmol,) into a three-necked flask containing 100 mL purified water. The solution is heated to 60 °C, upon which (0.800 g) 1 eq. of Cr­[NO_3_]_3_·9H_2_O dissolved in water ([Cr^III^] = 25 mM, 8 mL total), is added to the solution, resulting in a color change from colorless to green-colored [AlCr^III^W_11_O_39_]^6–^. The solution is heated under reflux (ca. 100 °C) and while vigorously stirring, 3 portions of K­[MnO_4_] (0.208 g total), summing to 0.66 eq. relative to Cr^III^ are added at 30 min intervals. The reaction was monitored by UV–Vis spectroscopy (Figure S1), which indicated oxidation of Cr^III^ in the POM to Cr^V^ and reduction of Mn^VII^ to Mn^IV^. The final pH-5, magenta-colored solution is optically transparent. As shown above (Figure 1 and related discussion), the reaction between [AlCr^III^W_11_O_39_]^6–^ and [MnO_4_]^−^ results in formation of μ-oxo bridges between the Cr­(V) atom in the POM and the ε-MnO_2_ core.

### Isolation and Purification of 1

To the magenta solution described above, KCl was added to a concentration of 2 M, resulting in precipitation of **1** as a dark brown solid. The solid was separated by centrifugation (15 min at 4000 rpm), after which the supernatant solution was decanted leaving a moist residue of solid **1**, which was readily redissolved in 15 mL of water to produce an optically transparent brown pH-7 solution. This process was carried out three or four times as needed to remove traces of byproducts, [AlCr^V^W_11_O_40_]^6–^and unreacted [MnO_4_]^−^, whose absence was confirmed by UV–Vis spectroscopy after redissolution of **1**. To remove added KCl salt, the solution was then placed in a regenerated-cellulose dialysis membrane (45 mm flat-width tubes; 12–14000D molecular-weight cutoff) and dialyzed against pure water in a 1 L beaker for 72 h, replacing the water once every 8 h. After an additional 48 h of dialysis, no K^+^ was detected by ICP-OES, indicating formation of the proton form of **1**. The method and values used to determine average ligation of 7.7 POM anions to each ε-MnO_2_ NC are summarized in Table S1.

### Assembly

Potassium cations were added to solutions of **1** at ratios of 8.8, 13.2, 17.6 equiv per POM complexing ligand, i.e., 0.13, 0.2, and 0.27 mM K^+^, respectively, and the solutions analyzed after 7 days by cryo-TEM (Figure S10). With increasing ratios of K^+^ per POM, the assemblies became more well-defined, eventually forming cubic assemblies 110 ± 20 nm on each side (Figure S23), each comprised of an analytically calculated ca. 28,000 individual complexes of **1**. Initial addition of a large excess of K^+^ (650 K^+^ per POM, equal to 10 mM K^+^) resulted in clumped aggregates of the cubic crystallites, but not in the formation of larger individual cubes (Figure S11).

### Time-Resolved Cryo-TEM Imaging

To a series of solutions containing **1** (3.34 μM) were added 1 mM solutions of KNO_3_ to give final K^+^:POM ratios of 1:17.6 (0.27 mM K^+^ to 1.99 μM of **1**). To arrest growth for cryo-TEM imaging, the solutions were cryogenically cooled immediately, and 5 min, 20 min, 1 h, 5 h, 24 h and 7 days after K^+^ addition. To maximize the homogeneous distribution of **1** and K^+^, each of the samples, except for the one sampled immediately, were mixed vigorously for several min before sampling. The vial that was sampled immediately was mixed using the micropipette, and then vigorously shaken for several seconds. See the Methods section in the Supporting Information for details on sampling and imaging.

### Atomic Molecular Dynamics Simulations

The MD simulations were performed using the NAMD^82^ software package, and the results were visualized using the VMD^83^ software package. The systems with periodic boundary conditions were simulated in an isothermal–isobaric (normal temperature and pressure) ensemble at T = 300 K, maintained by the Langevin dynamics with a damping coefficient of γ_Lang_ = 0.1 ps^–1^ and P = 1 bar. Long-range electrostatic interactions were evaluated using a particle mesh Ewald summation.^84^ TIP3P water parameters were assigned from the CHARMM36 ^85,86^ force field.

RDF gives the distribution of cations at different distances r from the center (Al atom) of the POM anion (Figure 4a):

$$g\left(r\right)=\underset{\Delta r\to 0}{\lim}\frac{\Delta p\left(r\right)}{4\pi \left(N_{pairs}/V\right)r^{2}\Delta r}$$ 1

Here, r is the distance between a pair of particles, Δp­(r) is the average number of atom pairs found at a distance between r and r + Δr, V is the total volume of the system, and Npairs is the number of unique pairs of atoms where one atom is from each of two sets (selections), sel1 and sel2.

The bulk van der Waals coupling of POM-NCs was described by the potential energy of two NCs MnO_2_ cores,

$$W_{ij}^{vdW}\left(dij\right)=-\frac{A}{12}\left\{\frac{R}{d_{ij}\left(1+\frac{d_{ij}}{4R}\right)}+\frac{1}{1+\frac{d_{ij}}{R}+\frac{d_{ij}^{2}}{4R^{2}}}+2\ln\left(\frac{d_{ij}\left(1+\frac{d_{ij}}{4R}\right)}{R\left(1+\frac{d_{ij}}{R}+\frac{d_{ij}^{2}}{4R^{2}}\right)}\right)\right\}$$ 2

where *d*_*ij*_ is the distance between the surfaces of MnO_2_ NCs, *A* is the Hamaker constant for core–core interaction in water (*A* ∼ 7 × 10^–20^ J), and *R* = 1.2 nm is the radius of the MnO_2_ core. Due to the fast decay of van der Waals coupling with the distance between the two NCs, we consider interaction only between the NC and its first neighbors.

## Supplementary Material



## Notes

Detailed experimental procedures, characterization methods, and data, atomistic MD calculations and additional figures and tables (PDF)
