# Supplementary material for: Ion-Size Controlled Non-Classical Crystallization of Metal-Oxide Nanoparticles Covered with a Few Highly Charged Ligands
Source: J Am Chem Soc. 2026 Jan 21;148(4):4373–84. doi: 10.1021/jacs.5c18213 (PMC12879739; doi:10.1021/jacs.5c18213)
Supplement: Supplementary file 1 [file ja5c18213_si_001.pdf]

Supporting Information for:

Ion-Size Controlled Non-Classical Crystallization of Metal-Oxide Nanoparticles Covered with a Few Highly Charged Ligands

Mark Baranov,[b,$] Jintumol Mathew,[c,$] Aranya Kar,[a] Nitai Leffler,[a] Arti Joshi,[a] Shubasis Roy,[a] Gal Gan-Or,[a] Vladimir Ezersky,[b] Petr Král,[c,d*] and Ira A. Weinstock[a,b*]

[a]Department of Chemistry, Ben-Gurion University of the Negev, Beer Sheva 84105, Israel.
[b]Ilse Katz Institute for Nanotechnology Science, Ben-Gurion University of the Negev, Beer-Sheva 84105, Israel.
[c]Department of Chemistry, University of Illinois Chicago, Chicago, Illinois 60607, USA.
[d]Department of Physics, Pharmaceutical Sciences, and Chemical Engineering, University of Illinois Chicago, Chicago, Illinois 60607, USA.

$M.B. and J.M. contributed equally to this paper
Email: pkral@uic.edu, iraw@bgu.ac.il

Table of Contents

Materials S3
Instrument methods S3
Synthesis and analytical methods S4
Fig. S1. Synthetic route for obtaining **1**. S18
Fig. S2. Size analysis of **1**. S19
Fig. S3. High resolution images of **1** and FFT analysis. S19
Fig. S4. EDS analysis of **1**. S20
Fig. S5. ESI-MS analysis of **1**. S20
Fig. S6. EPR analysis of liberated $[AlCr^{V}W_{11}O_{40}]^{6-}$ ions. S21
Table S1. POM coverage on the MnO_2 cores of **1**. S21
Fig. S7. XPS analysis of **1**. S22
Fig. S8. Cyclic voltammetry analysis of **1**. S22
Fig. S9. Zeta potential analysis of **1**. S23
Fig. S10. Cryo-TEM images after incremental titration of **1** with K^+. S24
Fig. S11. Cryo-TEM images after excess addition of K^+ to **1**. S25
Fig. S12. Cryo-TEM immediately after addition of K^+ to **1**. S25
Figs. S13-S15 and **Table 2**. Fractal analysis of **1** after K^+ addition. S26-28
Figs. S16-S21. Time resolved cryo-TEM of **1** after K^+ addition. S29-31
Fig. S22. Isothermal titration calorimetry (ITC) of branching-node formation and growth. S32
Table S3. Thermodynamic parameters for titration of K^+ into aqueous **1**. S32
Fig. S23. Size distribution of crystallites of **1**. S33
Fig. S24. Zeta potential values at various intervals after K^+ addition. S34
Fig. S25. DLS of **1** after addition of various monovalent cations. S34
Fig. S26. Computational modeling of the self assembly of **1**. S35
Fig. S27. DLS one week after addition of Li^+ to **1**. S36
Fig. S28. Cryo-TEM one week after addition of Li^+ to **1**. S36
References. S37

Materials

All materials were purchased as reagent grade and used without further purification. Chromium (III) nitrate nonahydrate ($Cr(NO_3)_3{\cdot}9H_2O$, Sigma-Aldrich, US), Sodium hydroxide (NaOH, flake, 98.9% assay, Alfa Aesar, England), KCl (analytical grade, Frutarom, Israel), Potassium bromide (KBr for IR spectroscopy, Fisher Chemical, UK), Tetrabutylammonium bromide (TBABr, 99+%, ACROS ORGANICS, USA), Tetraheptylammonium bromide (THABr, 99+%, ACROS ORGANICS, USA), Acetonitrile (99.8%, Alfa-Aesar), HCl (analytical grade, Bio Lab, Ltd., Israel), Sodium tungstate dihydrate ($Na_2WO_4{\cdot}2H_2O$, extra pure, Merck, US), Potassium permanganate ($KMnO_4$, 99%, Ridel-de Haën, Germany). Additional reagent-grade salts, acids and diethyl ether for polyoxometalate synthesis and reactions were obtained from commercial sources and used as received. Regenerated-cellulose dialysis membranes (45-mm flat-width tubes; 12−14000 Da MWCO) were purchased from VWR Scientific, treated before using to remove glycerin and traces of sulfur compounds, and stored in water at 5 °C. All water used for cleaning, synthesis and reactions was of high purity (18.2 M·Ω resistivity) from a Millipore® Direct-Q water-purification system. The polyoxometalate salt $K_9[\alpha\text{-}AlW_{11}O_{39}]{\cdot}13H_2O$, was prepared according to literary methods.[1] The purity of each POM salt was confirmed as appropriate by ^{27}Al NMR and FTIR.

Instrumental methods

UV-Vis spectra were recorded on a Hewlett-Packard 8453 diode array spectrophotometer.

pH values were measured using a Thermo SCIENTIFIC, ORION STAR A211 pH meter, or a EUTECH INSTRUMENTS, cyberscan pH 11 pH/ mv/ °C Meter. Prior to use, the pH meter was calibrated using standard reference solutions (pH 4.01, 7.00 and 10.01).

Dynamic light scattering (DLS) data were collected at 25 °C using two instruments: 1) ALV-CGS-8F instrument (ALV-GmbH, Germany). The regular angle of measurements was 90 degrees geometry of the detector with respect to incident beam (otherwise will be noted) and the CONTIN method was used to obtain hydrodynamic radii (Rh). Prior to each measurement, solutions were filtered through 0.22 μm polyvinylidene fluoride (PVDF) Millipore filters and diluted accordingly (to discharge the multiple scattering effect, which might distort the Rh results). 2) Malvern Zetasizer Nano S90, with size measurement from 0.3nm (diameter) to 5 microns using 90 degree scattering optics. Zetasizer software was used to obtain particle diameter. The DLS data were obtained to ensure that colloidal solutions of POM-protected metal-oxide NPs contained significant concentrations of particles in the size range of interest.

Zeta potential measurements were carried out by putting 1.0 mL of solution into measuring cell occupied with two electrodes. Zeta potential data were obtained using a ZEM 3600, Zetasizer, Malvern Instruments Ltd.

Electrospray ionization mass spectroscopy (ESI-MS) spectra were recorded from a LTQ Orbitrap XL instrument (Thermo Scientific, with an accuracy of 0.1 amu) with a nanospray ion source. TBA-salt of the POMs were used to make a solution in a mixture of 50:50 pure acetonitrile and pure water respectively and directly injected for the ESI-MS measurements.

X-ray diffraction (XRD) data was obtained using a Rigaku XtaLAB Synergy-S diffractometer using Cu Kα radiation (λ = 1.5405 Å), operated at 50 kV and 1 mA, equipped with a Hy-Pix-6000HE detector. Fourier-transform infra-red (FTIR) spectra were acquired using a Nicolet Impact 410 spectrophotometer (KBr pellets), analyzed with Omnic 7 software in transmission mode.

X-ray photoelectron spectroscopy (XPS) measurement was performed using an X-ray photoelectron spectrometer ESCALAB 250 ultrahigh vacuum ($1{\cdot}10^{-9}$ bar) apparatus with an Al K^{α} X-

ray source and a monochromator. The X-ray beam size was 500 μm and survey spectra was recorded with pass energy (PE) 150 eV and high energy resolution spectra were recorded with pass energy (PE) 20 eV. Processing of the XPS results was carried out using XPSPEAK program. **Energy dispersive x-ray spectroscopy (EDX)** analysis was done using a JEOL JEM- 2100F TEM operating at 200 kV equipped with a JED2300T energy dispersive X-ray spectrometer. JEOL Analytical Station software (v. 3.8.0.21) was used for the EDX data analysis.

Samples for dry TEM and high-resolution TEM (HR-TEM) and scanning transmission electron microscopy (STEM) were prepared by pipetting 5-10 μL of the aqueous sample solution onto Au grids covered with thin carbon-support films and dried in air. TEM data were obtained using a FEI Tecnai 12 G^2 electron microscope (120 kV) equipped with a Gatan slow-scan camera. HR-TEM data were obtained using a JEOL JEM-2100F instrument operated at an accelerating voltage of 200 kV.

Samples for cryogenic TEM (cryo-TEM) were prepared using a fully automated vitrification device ("Leica"). First, 3 μL of the sample solution were placed by pipette onto a glow discharged 300 Mesh Cu grid covered with a lacey-carbon film, held inside a 100% humidity chamber. The grid was then mechanically "blotted" and immediately plunged into liquid ethane (b.p. 185K) cooled by liquid nitrogen (b.p. 77K). Data were collected on the FEI Tecnai 12 G2 instrument (120 kV) and the Gatan slow-scan camera, 3 using a low-dose regime (to slow down the crystallization of vitrified water and to delay the formation of other artifacts due to beam damage). All images from both dry- and cryo-TEM (including electron diffraction patterns) were analyzed using Digital Micrograph Gatan Inc. software.

Induction coupled plasma optical emission spectroscopy (ICP-OES) was performed using a SPECTRO ACROS ICP-OES analyzer calibrated using standard solutions of the respective elements.

Electron paramagnetic resonance (EPR) analysis was perform using an EMX-220 CW EPR spectrometer operated at a frequency of 9.473 MHz. The samples were vacuum dried and inserted into a capillary tube, after which a spectrum was obtained and analyzed using WinEPR.

Cyclic voltammetry (CV) measurements of the $[\alpha\text{-AlCrVW}_{11}O_{40}]^{6-}$-complexed ε-MnO_2, **1** were performed using a CHI 760C potentiostat in a three-electrode cell at 25 ± 2 °C. A 2-mm diameter glassy carbon electrode served as the working electrode, while a Pt-wire and an Ag/AgCl (3M KCl) electrode were used as the counter and reference electrodes, respectively.

Isothermal titration calorimetry (ITC) was performed on a NANO ITC System (TA Instruments). Titrations were performed in pure water as reference at pH 7. Injections of 5 μl of 4.4 mM KCl were added from a computer-controlled microsyringe at an interval of 300s into a 3.34 μM complexes solution, with stirring at 300 r.p.m. at 25 °C. The experimental data were analyzed with NanoAnalyze software (TA Instruments) and were fitted to an independent model concurrently against a blank (addition of KCl to water) constant model to adjust for the heat of dilution.

Synthesis and analytical methods

Synthesis of $[\alpha\text{-AlCr}^{V}W_{11}O_{40}]^{6-}$ Complexed ε-MnO_2 cores and Colloidal MnO_2. A typical synthesis of **1** (Fig. S1, Top) involves the addition of $K_9[AlW_{11}O_{39}]\cdot 13H_2O$ (0.6518 g, 0.2 mmol,) into a three necked flask containing 100 mL Milli-Q water. The solution is heated to 60°C, upon which (0.800 g) 1 eq. of $Cr[NO_3]_3\cdot 9H_2O$ dissolved in water ($[Cr^{III}]$ = 25 mM, 8 mL total), is added to the solution; a color change from colorless to green is observed. The solution is heated under reflux to 100 °C while vigorously stirring and then 3 portions amounting to 0.66 eq. relative to Cr^{III} (0.208 g total) of $K[MnO_4]$ are added in 30 minute intervals, the reaction, which was followed by UV-Vis spectroscopy (Fig. S1, Bottom), involves the oxidation of Cr^{III} in the POM to Cr^{V} while reducing the

equivalent amount of Mn^{VII} to Mn^{IV} and covalently attaching via oxo bridges the POM to the ε-MnO_2 core, the final color is an optically transparent, magenta colored, pH 5 solution.

Isolation and Purification. In order to purify the product, 2 M KCl were added to the magenta solution which resulted in the precipitation of the complexes as dark brown solid - under these conditions, the ionic strength in the solution increases leading to reversible aggregation of the POM-capped MnO_2 cores which decreases their solubility in water, so that they can be separated from the supernatant solution by centrifugation (15 min at 4000 rpm), after which the supernatant was decanted leaving a moist residue of the complexes. After separation, the complexes were readily re-dissolved a small amount of pure water (15 mL) to produce an optically transparent brown pH-7 solution, this process was repeated several times to remove any byproducts, consisting of unreacted $[MnO_4]^-$ and $[AlCr^VW_{11}O_{40}]^{6-}$, until the UV-vis spectrum of the supernatant confirmed the sole presence of water. Next, the solution was placed in a cellulose membrane against pure water in a 1 L beaker for 72 hrs, replacing the water once every 8 hrs, to be sure that trace amounts of KCl salt and any POMs were no longer present in the solution. After an additional 48 h of dialysis, the pH was 5 and no K^+ was detected by ICP-OES, indicating formation of the proton form of **1**.

Yield (~20 mL solution after dialysis): 0.057 g, 46.2% based on Mn content from ICP-OES.

Synthesis of Colloidal MnO_2. Colloidal MnO_2 was synthesized by reacting $KMnO_4$ with S_2O_3 according to literary procedures[2] and verified using FTIR and UV-vis.

Synthesis of Molecular Polyoxometalate Containing Cr^{III} and Cr^V.

Synthesis of $[AlCr^{III}(H_2O)W_{11}O_{39}]^{6-}$. 0.1304 gr ($4\times10^{-5}$ mol) of $K_9[AlW_{11}O_{39}]\cdot13H_2O$ were dissolved in 20 ml water (pH after addition = 7.08), into which 1 eq. of Cr(III) was added from a stock solution of $Cr(NO_3)_3\cdot9H_2O$ ($[Cr^{III}]$ = 25 mM) while vigorously stirring the solution using a magnetic stirrer (pH after addition = 5.21), the solution was left to stir for an additional 60 minutes (final pH = 5.02). The solution was dried completely using a rotary evaporator. The material was re-crystallized by re-dissolving in a minimal amount of boiling water and left overnight at 4°C to give amorphous green material. IR (KBr pellet): γ_{as}(Al-O) 1079 cm^{-1}, γ_{as}(W=O) 976 cm^{-1}, γ_{as}(W-O$_{corner}$-W) 891 cm^{-1}, γ_{as}(W-O$_{edge}$-W) 814 cm^{-1}, 748 cm^{-1} (shoulder), 696 cm^{-1} (shoulder), 667 cm^{-1}. Yield (as $K_6[AlCr^{III}(H_2O)W_{11}O_{39}]$): 0.0891 g (74.8%).

Synthesis of $[AlCr^VW_{11}O_{40}]^{6-}$. In order to oxidize the Cr^{III} atom to Cr^V bulk electrolysis of solution containing $[AlCr^{III}(H_2O)W_{11}O_{39}]^{6-}$ (0.0595 g, 2×10^{-5} mol) at a potential of 1.6 V was performed over 3 hours, with the solution changing color from green to yellow, similarly to a procedure done by Rong et. al.[3] The material was isolated by addition of TBABr (tetrabutylammonium bromide) to precipitate the water insoluble $TBA_6[AlCr^VW_{11}O_{40}]$, yellow colored, salt. In order to verify that the POM indeed has a Cr^V atom an EPR measurement was performed, showing a single transition at 1.96 G, corresponding to a d^1 complex of Cr(V). IR (KBr pellet): 967 cm^{-1} γ_{as}(Al-O), 894 cm^{-1} γ_{as}(W=O), 798 cm^{-1} γ_{as}(W-O$_{corner}$-W) and 751 cm^{-1} γ_{as}(W-O$_{edge}$-W), 662 cm^{-1} (shoulder), 539 cm^{-1} (shoulder), 441 cm^{-1}. Subsequent investigation of the by-product of the synthesis of **1** were compared to product obtain herein. Yield (as $TBA_6[AlCr^VW_{11}O_{40}]$): 0.0439 g (52.4%).

Characterization of the reaction by-product, $[AlCr^VW_{11}O_{40}]$. The by-product left in the reaction solution was examined using spectroscopic and analytical methods. After decanting the centrifuged mixture of **1** (during the purification process), the magenta supernatant contained some POM that has not complexed into **1** and unreacted $[MnO_4]^-$. The POM was isolated by addition of TBABr to precipitate the water insoluble $TBA_6[AlCr^VW_{11}O_{40}]$ salt. The FT-IR spectrum (Fig. 1f in the text, blue line) shows the characteristic bands of the intact Keggin anion,[4] and matched those for the independently synthesized POM (described above), with the characteristic bands located at 967 cm^{-1}

γ_{as}(Al-O), 894 cm^{-1} γ_{as}(W=O), 798 cm^{-1} γ_{as}(W-O$_{corner}$-W) and 751 cm^{-1} γ_{as}(W-O$_{edge}$-W), 662 cm^{-1} (shoulder), 539 cm^{-1} (shoulder), 441 cm^{-1}.

The presence of Cr^V in the POM was identified using EPR spectroscopy (Fig. S6). An allowed transition at g = 1.96 ($\Delta S = \pm 1$) was observed, corresponding to the d^1 Cr^V atom present.[5] As Cr^V is an otherwise highly unstable chemical species, it can be concluded that it is stabilized in the "pocket" of the POM after reaction, as previous studies have shown.[6, 7] Moreover, a much weaker, formally forbidden transition at exactly double the g value (g = 3.92, $\Delta S = \pm 2$) was observed,[8, 9] which is direct evidence for a formation of a dimer species consisting of two $[AlCr^VW_{11}O_{40}]^{6-}$ units linked by a μ_2 bridging oxo, $[(AlCr^VW_{11}O_{39})_2\text{-}(\mu_2\text{-}O)]^{10-}$.

Assembly studies. A series of solutions containing **1** (3.34 μM complexes in 1 mL) and a fixed concentration of alkali metal cations were added (Li^+, Na^+, K^+, Rb^+ and Cs^+, 5 mM) DLS measurements indicate that the particles show a trend of increase in average size (Fig. S25), similar to previously reported ion-pairing interactions involving POMs and alkali metal cations,[10-12] forming large assemblies. In order to precisely quantify the ratio of the POMs to alkali metal cations several parameters were taken into consideration: the density of ε-MnO_2, the size of the individual NPs of **1**, the relative abundance of W to Mn and an estimated footprint of 1.8 nm^2 per POM, resulting in an average of 7.7 POMs per NC core (Table S1).

Next, using ICP-OES as a means of quantification, varying ratios of K^+ cations to POMs on the surface of the NCs were added (8.8, 13.2, 17.6 K^+ eq. per POM, equal to 0.13, 0.2 and 0.27 mM K^+, respectively), and observed using cryo-TEM. It was found that the greater the amount of potassium was added, the more well defined the assemblies became, eventually forming large cubic assemblies ca. 110 nm on each side, composed of up to an analytically calculated ca. 28,000 individual complexes of **1**. Large excess of K^+ (650 K^+ per POM, equal to 10 mM K^+) resulted in clumping of the observed cubes to form larger structures. Dialyzing the solution over a period of 3 days, exchanging the water every 12 hours, removed all K^+ ions and the solutions again contained individual complexes of **1**.

Assembly kinetics. A series of solutions containing **1** and a fixed ratio of POM:K^+ cations were prepared and sampled at varying time intervals (immediately after addition, 5 min, 20 min, 1 h, 5 h, 24 h and 1 week) to observe the formation steps of the BCC crystallites in situ. The stock solution of **1** (3.34 μM complexes) was mixed with a 1 mM solution of KNO_3 salt for a final ratio of POM:K^+ of 1:17.6 (1.99 μM of **1**, 0.27 mM K^+). To maximize the homogeneous distribution of **1** and K^+ ions in the solutions, each of the samples (except 0 minutes) were mixed vigorously using a stirrer for the appropriate amount of time before sampling. The 0 minutes sample was mixed using the micro-pipette, vigorous shaking for several seconds followed by a few second of vigorous stirring using a stirrer before sampling.

Calculation of the SL packing from profile analysis of cryo-TEM images. SL morphology and dimensions. TEM imaging reveals that the individual NC building blocks, including the POM ligands, are approximately 5 nm in diameter. At the superlattice scale, the assemblies exhibit approximately isotropic dimensions along three mutually perpendicular directions, which we can associate with [100], [010], and [001] of the SL. In other words, the SL extends to a similar degree along three orthogonal axes, giving rise to an overall cubic morphology with an implied inversion center. From a symmetry standpoint, this behavior is most naturally consistent with a cubic space group.

Cubic symmetry alone does not enforce isotropic crystal growth, and several cubic, non-centrosymmetric phases form highly anisotropic 1D morphologies. It is also important to acknowledge that even in high-symmetry cubic groups, crystals can grow anisotropically due to facet-dependent kinetics. As such, equal or nearly equal dimensions along [100], [010] and [001] are supporting rather than definitive evidence for a specific centrosymmetric cubic space group (i.e. FCC *Fm-3m*, BCC *Im-*

3m, SC *Pm-3m*). What they do strongly support is that the average SL structure has a cubic metric, and that high-symmetry cubic arrangements, such as BCC-type packings or closely related variants, are more natural candidates than low-symmetry or strongly anisotropic ones. In this context, the fact that our system does not show pronounced anisotropy but instead grows approximately isotropically along three equivalent ⟨100⟩ directions is a non-trivial and supportive observation for a simple cubic metric at the SL level. The specific cubic phase was determined as follows.

Density profile and oscillation period. Quantitative structural information from real space, was extracted from the intensity profile from a TEM image along a direction approximately parallel to a [100]-type projection of the SL. This profile reflects the projected density of the NCs and their surrounding POM ligands. It exhibits a series of oscillations: maxima correspond to regions of high core density, and minima to regions between these high-density planes.

We therefore proceeded conceptually as follows:

1) **Local maxima detection**. We first identified all local maxima in the intensity profile.
2) **Removal of shoulders/noise peaks**. Some local maxima are weak shoulders located very close to a nearby, much more intense maximum. Such shoulders are unlikely to represent distinct structural planes. We therefore sorted the maxima in increasing position x, imposing a minimum spacing threshold of roughly 1.5 nm.
3) **Spacing of "true" maxima**. We obtained a set of "true" maxima that capture the dominant structural modulation.

The spacing between successive cleaned maxima along the profile, Δx, was then examined. A clear cluster of spacings emerged around an average $\langle \Delta x \rangle = 2.84\, nm$ after removal of 3 larger gaps attributable to missing or strongly broadened peaks at longer distances (which is expected in a *dynamic, solvated* system). This average $\langle \Delta x \rangle$ is a real-space quantity. Obtained directly from TEM, and it provides a strict constraint on any proposed lattice.

Ideal BCC geometry vs. observed period. We now compare this experimental period with the expected behavior of an ideal SL with several NC packing organizations.

BCC lattice. As shown in the paper, we treat the NC as having an effective radius of ≈ 2.5 nm (i.e., a 5 nm core plus the POM size). In an ideal BCC lattice of touching spheres, the nearest neighbors lie along the body diagonal, and the following geometric relation holds for the lattice constant, *a*:

$$4r = \sqrt{3} \cdot a \Rightarrow a = \frac{4r}{\sqrt{3}}$$

$$r = 2.5\, nm \rightarrow a = \frac{10\, nm}{\sqrt{3}} = 5.8\, nm$$

For a [100] projection of a BCC lattice, planes of core centers (from corners and body centers of adjacent unit cells) appear at:

$$x = 0, \frac{a}{2}, a, \frac{3a}{2}, etc.$$

The expected spacing between successive core-density maxima along such a direction is therefore:

$$\Delta x_{BCC,[100]} = \frac{a}{2} = 2.9\, nm$$

This value is in excellent agreement with the experimentally observed oscillation period of the cleaned maxima:

$$\langle \Delta x_{exp.} \rangle = 2.84\, nm \approx \Delta x_{BCC,[100]}$$

In other words, the periodic modulation of the experimental TEM line profile is quantitatively consistent with a BCC-type arrangement of ~5 nm NCs viewed along a [100]-like direction. This is a non-trivial agreement, because, as we show immediately below, other plausible lattice types do not reproduce this period.

Consider the natural alternative packings for 5 nm particles, focusing on the expected interplanar or core-layer spacings along simple projections. We emphasize that this comparison uses only basic geometry; no fitting or parameter-tuning is involved.

FCC lattice. For a close-packed FCC lattice of particles with effective radius r:

$$4r = \sqrt{2} \cdot a \Rightarrow a = \frac{4r}{\sqrt{2}} = 2\sqrt{2}r$$

$$a = 2\sqrt{2} \cdot 2.5\, nm = 7.07\, nm$$

Along [100], strong planes of core centers occur at 0 and $\frac{a}{2}$, so the characteristic spacing is:

$$\Delta x_{FCC,[100]} = \frac{\mathrm{a}}{2} = 3.53\, nm$$

This value is already significantly larger than the experimental 2.84 nm. Along [110] or [111], the relevant distances are even larger (5 nm and above). Thus, an FCC SL of 5 nm particles does not reproduce the observed real-space modulation period.

Simple cubic (SC). For a simple cubic packing of particles of radius r, the lattice parameter is simply:

$$a = 2r = 5.0\, nm$$

Successive core layers along [100] are then spaced by a = 5.0 nm, again incompatible with the experimentally determined period.

Hexagonal close packed (HCP). For an ideal hexagonal close-packed lattice with sphere radius r = 2.5 nm, the in-plane parameter is:

$$a = 2r = 5.0\, nm$$

$$\frac{c}{a} = 1.633 \rightarrow c = 8.17\, nm$$

The natural interplanar spacings in such a structure (in-plane along a, $\frac{\sqrt{3}}{2}a$ and along the stacking direction [0001]) are in the 4-5 nm range. None of the simple HCP directions yields a dominant modulation around 2.8-2.9 nm.

Rutile-like tetragonal. Similarly, a reasonable tetragonal packing of 5 nm particles (with lattice constants a and c comparable to or larger than 2r) produces interplanar spacings of order 4-5 nm or more along high-symmetry directions. To obtain a primary layering at ~2.9 nm, one must essentially

“squeeze” the lattice along c, or to construct a “half-lattice” spacing reminiscent of the BCC $\frac{a}{2}$ modulation. Such a model becomes, in practice, a re-labelling of the same geometric situation that BCC already captures in a simpler, more natural way.

Taken together, this geometric analysis shows that for our SL, BCC naturally produces a short-range spacing of $\frac{a}{2} \approx 2.9\ nm$, and that FCC, SC, HCP, and straightforward tetragonal packings are all ruled out as they yield characteristic spacings of ≥ 3.5-5 nm along simple projections.

Despite the close fitting of the profile analysis with BCC, the crystal itself is imperfect due to:

1) Positional disorder (thermal fluctuations, finite correlation lengths).
2) Dynamic motion of particles and ligands.
3) Ligand shells of finite thickness and possible inhomogeneity.
4) Size and shape polydispersity of the NCs.

As a result, peaks in the experimental line profile are broadened, and a few maxima are missing, leading to occasional larger gaps between peaks. These deviations from the ideal pattern are expected in a *dynamic, solvated SL* and thus we do not claim a defect-free BCC crystal.

Electrostatic and thermodynamic basis of K^+ induced self-limiting superlattice assembly

1. Experimental parameters and constants

Parameter	Value	Source / assumption
Nanocrystal concentration	1.78 µM	Experimental
Potassium concentration	0.27 mM (as KNO_3)	Condition for stable SLs
High-salt condition	10 mM KNO_3	Control (aggregation of SLs)
Effective NC charge q_{NC}	−7.5 e	Calculated (see below)
NC radius, r_{NC}	2.5 nm	TEM
NC number density, n_{NC}	$3.8 \cdot 10^{16} \left[\frac{1}{m^3}\right]$	Calculated (see below)
Mean NC-NC spacing, d_{NC-NC}	$3\ [\mu m]$	Calculated (see below)
POM ligand charge	−1 e per ligand	Structural / chemical
Post-dialysis pH	$\approx 5\ \rightarrow\ [H^+] = 1 \cdot 10^{-5}[M]$	Experimental
Ionic strength at 0.27 mM KNO_3	$I\ =\ 3.25 \cdot 10^{-4}\ [M]$	Calculated (see below)
Ionic strength at 10 mM KNO_3	$I\ =\ 0.010\ [M]$	Calculated (see below)
Debye length at 0.27 mM	$\lambda_D = 17\ [nm]$	Calculated (see below)
Debye length at 10 mM	$\lambda_D\ =\ 3.0\ [nm]$	Calculated (see below)
Superlattice (SL) edge length, L_{SL}	110 nm	Cryo-TEM
Net SL charge, $q_{SL\ eff}$	−130 e	Calculated (see below)
SL number density, n_{SL}	$3.8 \cdot 10^{16} \left[\frac{1}{m^3}\right]$	Calculated (see below)
Mean SL-SL spacing, d_{SL-SL}	$3\ [\mu m]$	Calculated (see below)
ΔH	$-33.6\ \left[\frac{kcal}{mol}\right]$	ITC
−TΔS	$30.2\ \left[\frac{kcal}{mol}\right]$	ITC
ITC free energy, ΔG_{ITC}	$-3.4\ \left[\frac{kcal}{mol}\right]$	ITC
Temperature, T	298 K	Experiment
Vacuum permittivity, ε_0	$8.854 \cdot 10^{-12} \left[\frac{F}{m}\right]$	Physical constant
Water dielectric, ε_r	78.5	Physical constant at 298 K

Boltzmann constant, k_B	$1.38065 \cdot 10^{-23} \left[\frac{J}{K}\right]$	Physical constant
Elementary charge, e	$-1.602 \cdot 10^{-19}\ [C]$	Physical constant
Avogadro constant, N_A	$6.022 \cdot 10^{23} \left[\frac{1}{mol}\right]$	Physical constant
Faraday constant, F	$96485 \left[\frac{C}{mol}\right]$	Physical constant

2. Electrostatics in the single NC regime and comparison to ITC data

2.1. Nanocrystal number density and mean spacing.

The nanocrystal molar concentration is: $[NC] = 1.78 \cdot 10^{-6} \left[\frac{mol}{L}\right]$.

Thus:

$$c_{NC} = 1.78 \cdot 10^{-6} \left[\frac{mol}{L}\right] \cdot 1000 \left[\frac{L}{m^3}\right] = 1.78 \cdot 10^{-3} \left[\frac{mol}{m^3}\right].$$

The number density of nanocrystals is:

$$n_{NC} = c_{NC} \cdot N_A = 1.78 \cdot 10^{-3} \left[\frac{mol}{m^3}\right] \cdot 6.022 \cdot 10^{23} \left[\frac{1}{mol}\right] = 1.07 \cdot 10^{21} \left[\frac{1}{m^3}\right].$$

The mean center-to-center distance between NCs (for a random 3D distribution) is approximated by:

$$d_{NC-NC} = \sqrt[3]{n_{NC}} = \sqrt[3]{1.07 \cdot 10^{21} \left[\frac{1}{m^3}\right]} = 1.02 \cdot 10^{7} \left[\frac{1}{m}\right] = 9.8 \cdot 10^{-8} [m] = 98\ [nm]$$

This confirms that nanocrystals are initially well separated relative to their size and to the Debye length.

2.2. Ionic strength and Debye length at 0.27 mM KNO_3.

KNO_3 dissociates as $K^+ + NO_3^-$ (1:1 electrolyte). At $[K^+] = 0.27$ [mM]:

$$[K^+] = [NO_3^-] = 0.27 \cdot 10^{-3}\ [M] = 2.7 \cdot 10^{-4}\ [M].$$

The contribution of the NCs

The ionic strength for a 1:1 electrolyte is:

$$I = \frac{1}{2} \sum c_i z_i^2 = \frac{1}{2} \left(c_{K^+} z_{K^+}^2 + c_{NO_3^-} z_{NO_3^-}^2\right)$$

$$= \frac{1}{2}(2.7 \cdot 10^{-4}[M] \cdot 1^2 + 2.7 \cdot 10^{-4}[M] \cdot 1^2) = 2.7 \cdot 10^{-4}[M].$$

And from the NCs themselves:

$$I_{NC} = \frac{1}{2}[NC] \cdot z^2 = \frac{1}{2} \cdot 1.78 \cdot 10^{-6}[M] \cdot (-7.5)^2 = 5 \cdot 10^{-5}[M]$$

A small additional contribution from H^+ at pH ≈ 5 ($[H^+] = 1 \cdot 10^{-5}[M]$) is:

$$I_{H^+} = \frac{1}{2}[H^+] \cdot z^2 = \frac{1}{2} \cdot 1 \cdot 10^{-5}[M] = 5 \cdot 10^{-6}[M]$$

Thus the total ionic strength is $I = 3.25 \cdot 10^{-4}[M]$.

The Debye length λ_D is given by:

$$\lambda_D = \sqrt{\frac{\varepsilon^0 \cdot \varepsilon_r \cdot k_B \cdot T}{2\, N_A \cdot e^2 \cdot I \cdot 1000}}$$

Substituting numerical values at 298 K and $I = 2.7 \times 10^{-4}[M]$:

$\varepsilon^0 = 8.854 \cdot 10^{-12} \left[\frac{F}{m}\right] = 8.854 \cdot 10^{-12} \left[\frac{C^2}{m \cdot J}\right]$,

$\varepsilon_r = 78.5$,

$k_B = 1.38065 \cdot 10^{-23} \left[\frac{J}{K}\right]$,

$T = 298\, K$,

$N_A = 6.022 \cdot 10^{23} \left[\frac{1}{mol}\right]$,

$e = -1.602 \cdot 10^{-19}\, [C]$

The numerator is:

$$\varepsilon^0 \cdot \varepsilon_r \cdot k_B \cdot T = 8.854 \cdot 10^{-12} \left[\frac{C^2}{m \cdot J}\right] \cdot 78.5 \cdot 1.38065 \cdot 10^{-23} \left[\frac{J}{K}\right] \cdot 298\, K = 2.86 \cdot 10^{-30} \left[\frac{C^2}{m}\right]$$

The denominator is:

$$2N_A \cdot e^2 \cdot I \cdot 1000 = 2 \cdot 6.022 \cdot 10^{23} \left[\frac{1}{mol}\right] \cdot (-1.602 \cdot 10^{-19}[C])^2 \cdot 3.25 \cdot 10^{-4} \left[\frac{mol}{L}\right] \cdot 1000 \left[\frac{L}{m^3}\right] = 1.00 \cdot 10^{-14} \left[\frac{C^2}{m^3}\right]$$

Thus:

$$\lambda_D = \sqrt{\frac{2.86 \cdot 10^{-30} \left[\frac{C^2}{m}\right]}{1.00 \cdot 10^{-14} \left[\frac{C^2}{m^3}\right]}} = 1.7 \cdot 10^{-8}\, [m] = 17\, [nm]$$

2.3. Screened potential of a single nanocrystal.

The effective charge on each nanocrystal is given by $q = 4\pi \cdot \varepsilon_r \cdot \varepsilon_0 \cdot r \cdot \xi$, with NC radius $r = 2.5\, [nm] = 2.5 \cdot 10^{-9}\, [m]$, and zeta potential $\xi = -0.055\, [V]$.

Thus, the effective charge of each NC is:

$$q_{NC} = 4\pi \cdot 78.5 \cdot 8.854 \cdot 10^{-12} \left[\frac{C}{m \cdot V}\right] \cdot 2.5 \cdot 10^{-9}[m] \cdot -0.055\, [V] = -1.20 \cdot 10^{-18}\, [C]$$

Which also equals to an effective NC charge of $q_{NC} = -7.5\, e$, or approx. -1 per POM ligand (some protons screen the charge).

We model the NC as a screened point charge in Debye-Hückel approximation:

$$\varphi_{NC(r)} = \frac{q_{NC}\, e^{-\frac{r}{\lambda_D}}}{4\pi\, \varepsilon_0 \varepsilon_r\, r}$$

At the mean interparticle spacing $r = d_{NC-NC} = 98\ [nm]$:

$$\frac{r_{bulk}}{\lambda_D} \approx \frac{98[nm]}{17[nm]} = 5.8$$

Thus:

$$e^{-\left(\frac{r_{bulk}}{\lambda_D}\right)} = e^{-5.8} = 3.0 \cdot 10^{-3}$$

Substituting into $\varphi_{NC(r)}$, one obtains:

$$\varphi_{NC(r)} = \frac{-1.20 \times 10^{-18}\ [C] \cdot 3.0 \cdot 10^{-3}}{4\pi \cdot 78.5 \cdot 8.854 \cdot 10^{-12} \left[\frac{C}{m \cdot V}\right] 98\ [nm]} = -4.2 \cdot 10^{-6}[V]$$

$$\varphi_{bulk} = -4.2 \cdot 10^{-6}[V] = -4.2\ [\mu V]$$

which is effectively zero, a K^+ ion in bulk solution experiences negligible potential from a given NC.

Yet, one must consider the binding distance, calculated from MD, with major peaks at 0.735 [nm] and 0.845 [nm].

With $\lambda_D = 17\ nm$, $\frac{r_{bind}}{\lambda_D} = \frac{0.735[nm]\ or\ 0.845[nm]}{17\ [nm]} = 0.043\ or\ 0.050$ both give $e^{-\left(\frac{r_{bind}}{\lambda_D}\right)} \approx 0.96$. Substituting into the Debye-Hückel expression with the r_{bind} values, we obtain numerically that the potential ranges from -0.179 [V] to -0.156 [V].

The initial potential drop experienced by a K^+ ion moving from bulk to a binding site is then:

$\Delta\varphi_{before\ SL\ formation} = \varphi_{bind} - \varphi_{bulk} \approx -0.179[V] - 0[V] = -0.179[V]$ for the closer binding distance and $-0.156[V]$ for the longer binding distance, respectively.

2.4. Comparison to ITC ΔG.

The ITC-derived free energy of binding is:

$$\Delta G_{ITC} = -3.4 \left[\frac{kcal}{mol}\right]$$

Converting to SI, $1 \left[\frac{kcal}{mol}\right] = 4184 \left[\frac{J}{mol}\right]$:

$$\Delta G_{ITC} = -3.4 \cdot 4184 \left[\frac{J}{mol}\right] = -1.42 \cdot 10^{4} \left[\frac{J}{mol}\right]$$

For a monovalent ion (z = +1) and electrochemical theory with $\Delta G = z \cdot F \cdot \Delta\varphi$, the effective potential drop is:

$$\Delta\varphi_{ITC} = \frac{\Delta G_{ITC}}{z \cdot F} = \frac{-1.42 \cdot 10^{4} \left[\frac{J}{mol}\right]}{96485 \left[\frac{C}{mol}\right]} = -0.147\ V.$$

In comparison, the calculation above corresponds to $\Delta G_{calc.} = -3.6 \left[\frac{kcal}{mol}\right]$ $and - 4.1 \left[\frac{kcal}{mol}\right]$ A slightly larger effective distance (~0.87-0.90 [nm]) or a slightly less negative effective charge (e.g. -6.5 to -7 e) would make the model land **exactly** on $-3.4 \left[\frac{kcal}{mol}\right]$.

Within MD uncertainty and modest model approximations, the continuum electrostatics reproduces the ITC ΔG essentially quantitatively.

3. Electrostatics in the superlattice regime

3.1. Superlattice concentration and spacing.

In the self-limiting regime, nanocrystals assemble into cubic BCC superlattices (SLs) containing $\frac{N_{NC}}{SL} = 28{,}000\ NCs.$ Given $[NC] = 1.78 \cdot 10^{-6} \left[\frac{mol}{L}\right]$, the SL molar concentration is:

$$[SL] = \frac{[NC]}{28000} = \frac{1.78 \cdot 10^{-6} \left[\frac{mol}{L}\right]}{28000} = 6.36 \cdot 10^{-11} \left[\frac{mol}{L}\right]$$

$$c_{SL} = 6.36 \cdot 10^{-11} \left[\frac{mol}{L}\right] \cdot 1000 \left[\frac{L}{m^3}\right] = 6.36 \cdot 10^{-8} \left[\frac{mol}{m^3}\right]$$

The SL number density is:

$$n_{SL} = c_{SL} \cdot N_A = 6.36 \cdot 10^{-8} \left[\frac{mol}{m^3}\right] \cdot 6.022 \cdot 10^{23} \left[\frac{1}{mol}\right] = 3.83 \cdot 10^{16} \left[\frac{1}{m^3}\right]$$

The corresponding mean center-to-center SL spacing:

$$d_{SL-SL} = n_{SL} = \sqrt[3]{3.83 \cdot 10^{16} \left[\frac{1}{m^3}\right]} \to 3.0 \cdot 10^{-6}[m] = 3\ [\mu m]$$

3.2. Screened potential of a weakly charged superlattice.

Assuming and equal radius sphere to the volume of a 110 [nm] superlattice, and using the measured zeta potential of $\xi = -0.035\ [V]$ shows that almost all internal POM charge is neutralized by K^+ and H^+, leaving a residual net charge per superlattice of:

$$V_{SL} = (110\ [nm])^3 = 1{,}331{,}000[nm^3]$$

$$V_{equivalent\ sphere} = \frac{4}{3}\pi r^3 \to r_{equivalent\ sphere} = \sqrt[3]{\frac{3}{4}\frac{V_{equivalent\ sphere}}{\pi}}$$

$$r_{equivalent\ sphere} = \sqrt[3]{\frac{3}{4} \cdot \frac{1{,}331{,}000[nm^3]}{\pi}} = 68.2\ [nm] = 6.82 \cdot 10^{-8}[m]$$

$$q_{SL} = 4\pi \cdot 78.5 \cdot 8.854 \cdot 10^{-12} \left[\frac{C}{m \cdot V}\right] \cdot 6.82 \cdot 10^{-8}[m] \cdot -0.035\ [V] = -2.08 \cdot 10^{-17}\ [C]$$

Thus $q_{SL\,eff} = -130\ e.$

The logical location of this effective charge is at the SL surface, thus we need to calculate the amount of NCs at the surface and obtain the effective charge per NC at the surface for a SL with a side length of 110 [nm] and NC diameter of 5 [nm] (including the core and ligands).

An NC is considered "interior" if its center can lie anywhere within $(L-d)^3$, i.e. any center that is at least one NC radius from each face, so the interior-accessible cube is given by:

$$L_{interior} = L - d = 110[nm] - 5[nm] = 105[nm]$$

Ratio of interior volume to full volume:

$$f_{interior} = \left(\frac{L_{interior}}{L}\right)^3 = \left(\frac{105[nm]}{110[nm]}\right)^3 = (0.955)^3 = 0.871$$

So, about 87.1% of the NCs are in the interior of the SL:

$$NC_{interior} = 0.871 \cdot 28000 = 24388$$

$$NC_{exterior} = NC_{SL} - NC_{interior} = 28000 - 24388 = 3612$$

Thus the effective charge on each exterior NC is:

$$\frac{q_{SL\,eff}}{NC_{exterior}} = \frac{-130}{3612} = -0.036$$

This results in a much lower effective charge compared to the initial -7.5 effective charge per NC.

Next, we need to calculate the potential drop at various distances and compare it with the previously obtained values for a single NC.

The ionic strength contribution of the superlattice is:

$$I_{SL} = \frac{1}{2}[SL] \cdot z^2 = \frac{1}{2} \cdot 6.36 \cdot 10^{-11}[M] \cdot (-130)^2 = 5.4 \cdot 10^{-7}[M]$$

Which is essentially negligible, thus the effective ionic strength stems from the K^+, NO_3^- and protons. Since the pH practically does not change after SL formation (meaning there are still many protons bound to the POMs / NC surface), the ionic strength contribution of the protons is the same as before.

The superlattices do uptake a certain amount of K^+ from solution, as there are ~28000 particles and only an effective charge of -130 per SL. One needs to also consider that the effective charge on each NC was -7.5, thus only 7.5 K^+ are needed to neutralize each particle:

$$28000 \left[\frac{NC}{SL}\right] \cdot 7.5 \left[\frac{K^+}{NC}\right] = 209{,}870 \left[\frac{K^+}{SL}\right]$$

Total uptake of K^+:

$$6.36 \cdot 10^{-11} \left[\frac{mol_{SL}}{L}\right] \cdot 6.022 \cdot 10^{23} \left[\frac{SL}{mol}\right] \cdot 209{,}870 \left[\frac{K^+_{uptake}}{SL}\right] = 8.04 \cdot 10^{18} \left[\frac{K^+}{L}\right]$$

$$\frac{8.04 \cdot 10^{18}\left[\frac{K^+}{L}\right]}{6.022 \cdot 10^{23}\left[\frac{K^+}{mol}\right]} = 1.33 \cdot 10^{-5}\left[M_{K^+ uptake}\right]$$

Thus after the uptake, the concentration of K$^+$ is now $2.567 \cdot 10^{-4}\left[M_{K^+ free}\right]$.

The ionic strength of the solution is:

$$I = \frac{1}{2}\sum c_i z_i^2 \ = \frac{1}{2}\left(c_{K^+} z_{K^+}^2 + \ c_{NO_3^-} z_{NO_3^-}^2 + c_{H^+} z_{H^+}^2\right)$$

$$I = \frac{1}{2}(2.567 \cdot 10^{-4}[M] + 2.7 \cdot 10^{-4}[M] + 5 \cdot 10^{-5}[M]) = 2.68 \cdot 10^{-4}[M]$$

The Debye length is now $\lambda_D \ = 18.5\ [nm]$, calculated as in the previously shown fashion.

The screened potential of a superlattice with $q_{SL} = -130$ at distance r is given in the same Debye-Hückel form:

$$\varphi_{SL(r)} \ = \frac{q_{SL}\ e^{-\frac{r}{\lambda_D}}}{4\pi\ \varepsilon_0 \varepsilon_r\ r}$$

Taking $\lambda_D \ = 18.5\ [nm]$ and evaluating several distances for $\varphi_{SL(r)}$ shows that the potential drops are much lower than previously. For example, at $r \ = \ 50\ [nm]$, one finds $\varphi_{SL} \ = -3 \cdot 10^{-3}[V]$, at $r \ = \ 100\,[nm],\ \varphi_{SL} = \ -1 \cdot 10^{-4}[V]\ (0.1\,mV),$ and at $r \ = \ 1\ [\mu m],\ \ \varphi_{SL} \approx -10^{-26}[V]$.

In other words, on the micrometre length scale separating distinct SLs, the electrostatic potential generated by a superlattice is effectively zero.

For a single NC on the surface with an effective charge of only -0.036, the same applies, even at the binding distances calculated from MD, with $\varphi_{NC_{exterior}}$ values of -0.861 [mV] and -0.744 [mV] for 0.745 [nm] and 0.845 [nm], respectively.

For a K$^+$ ion in bulk solution between superlattices, the potential difference between any two locations separated by several microns is negligible:

$$\left|\Delta\varphi_{final}\right| \ll 1\ [mV] \rightarrow \left|\Delta G_{final}\right| \ = zF\left|\Delta\varphi_{final}\right| \ll 0.01\left[\frac{kcal}{mol}\right] \ll k_B T$$

Thus, in the final SL regime there is essentially no electrostatic driving force for additional K$^+$ association or for adding further NCs to a given SL.

One must also consider the repulsion interaction between two adjacent SLs at various concentrations of K$^+$, as different concentrations lead to different screening of the SL charge of -130 using the screened Coulomb (Debye-Hückel) interaction energy equation:

$$U(r) = \frac{q^2}{4\pi\varepsilon_0\varepsilon_r}\frac{e^{\frac{r}{\lambda_D}}}{r}\quad [J]$$

Using $q \ = \ 2.08 \cdot 10^{-17}[C]$ and a touching distance between the cubes r = 110 [nm] (center to center), one obtains $U(r) \ = 1.1 \cdot 10^{-21}[J]$ for a concentration of 0.27 [mM] K$^+$, and $U(r) \ = 5.4 \cdot 10^{-35}[J]$ for a concentration of 10 [mM] (after calculating for $\lambda_D \ = 3\ [nm]$ given the

substantially increased ionic strength), a 14 order of magnitude difference, thus the repulsion between the SLs at higher concentrations is substantially weaker.

Summary. These calculations permit a quantitative explanation of the self-limiting superlattice assembly. In the initial dispersed regime, each nanocrystal carries an effective charge of -7.5 e, and the Debye length at 0.27 mM KNO_3 + 1.78 μM NC solution is $\lambda_D = 17$ nm. The NCs are roughly 98 nm apart, so a K^+ ion in bulk solution experiences a potential that is effectively zero. When a K^+ approaches a NC and binds one hydration shell from the POM-decorated surface, it experiences a potential drop of -0.179 V or -0.155 V, based on K^+ positions determined by MD simulation, corresponding to $\Delta G_{calc.} = -4.1 \left[\frac{kcal}{mol}\right] and - 3.6 \left[\frac{kcal}{mol}\right]$, respectively, in very good agreement with the ITC measurement.

After K^+ is added and the NCs assemble, K^+ association in the interior of the forming SLs reduces its net charge to only -130e per SL. At the same time, the NC population is partitioned into relatively few SLs, with a mean SL-SL separation of ~3 μm. With the Debye length being even larger at 18.5 nm, the electrostatic potential of a SL decays to negligible values ($\ll$ 1 mV), and is vanishingly small between SLs at micrometer separations. This holds true for NCs on the SL surface as well.

In this final regime, the free energy change for bringing an additional K^+ from bulk into the vicinity of a SL, or for adding an extra NC to an already formed lattice, approaches very small. Any further growth would provide negligible additional electrostatic stabilization while incurring entropic penalties. This leads to a finite equilibrium aggregation number of approx. 28,000 nanocrystals per SL, conceptually analogous to the thermodynamically controlled number of amphiphiles in micelles at the critical micelle concentration.

At a much higher salt (10 mM KNO_3), the Debye length shrinks to $\lambda_D = 3$ nm. The field around individual NCs and SLs becomes highly localized, and K^+ screening is essentially complete at nanometer distances. Under these conditions, residual attractive forces and ion-bridging effects dominate over long-range repulsion, and the SLs aggregate while retaining their original pre-programmed 110 nm size, as shown by cryo-TEM (Figure S11). This behavior is fully consistent with the electrostatic framework used here.

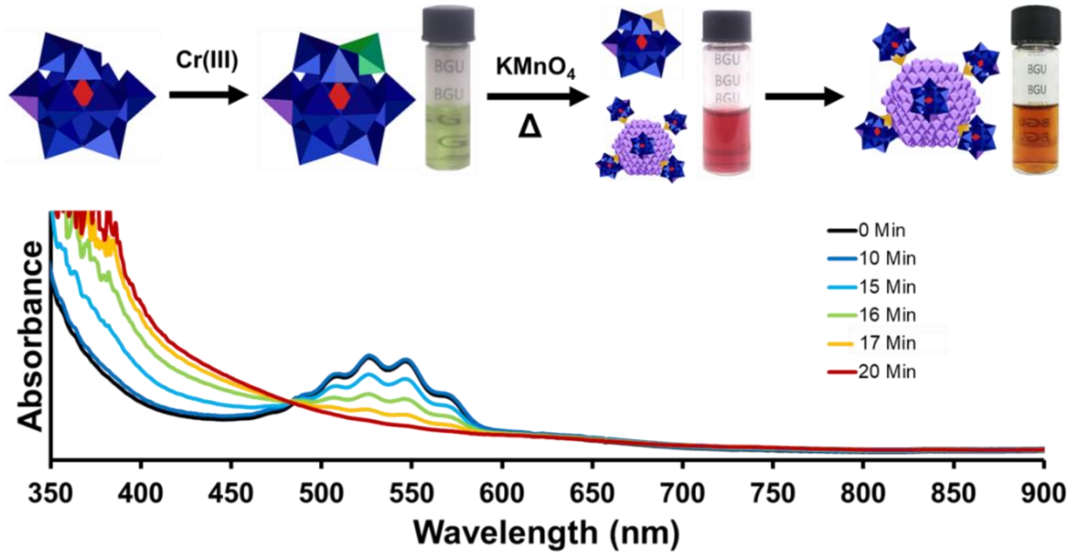

Fig. S1. Top: Synthesis of POM-complexed ε-MnO_2 (**1**). To a solution containing $[\alpha\text{-}AlW_{11}O_{39}]^{9-}$, 1 eq. of Cr^{III} was added to produce a clear green solution of $[\alpha\text{-}AlCr^{III}W_{11}O_{39}]^{6-}$. The Cr^{III} complex was then reacted with $[MnO_4]^-$ at 100°C (reflux at 1 atm ambient pressure), at a stoichiometry of 2:3 Mn^{VII} to Cr^{III}, for 1.5 hours, yielding a clear magenta solution containing the product, **1**, and reaction by-products $[\alpha\text{-}AlCr^{V}W_{11}O_{40}]^{6-}$ and unreacted $[MnO_4]^-$. The reaction solution was treated with KCl and centrifuged, after which the precipitate was re-dissolved in pure water. This was carried out three times, after which dialysis against pure water for 72 hours gave a clear brown solution of **1**. **Bottom**: UV-Vis spectrum obtained during the redox reaction between $[\alpha\text{-}AlCr^{III}W_{11}O_{39}]^{6-}$ and $[MnO_4]^-$. Over 20 min, absorbance at the distinct $[MnO_4]^-$ fingerprint region between 475-600 nm decreases in concert with a increase in absorbance at 350-475 nm. Color code: blue octahedra WO_6, green octahedra – $Cr^{III}O_6$, yellow octahedra – $Cr^{V}O_6$, purple octahedra – $Mn^{IV}O_6$, red tetrahedra – PO_4.

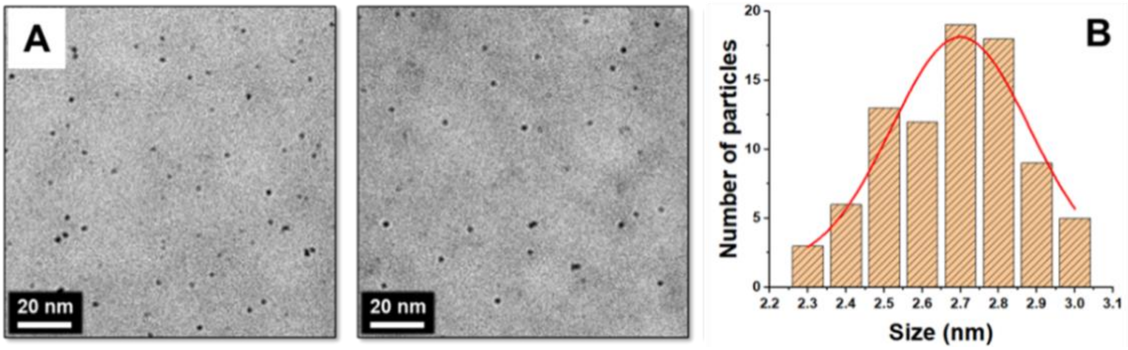

Fig. S2. Cryo-TEM and size analysis of individual complexes of 1. (A) Cryo-TEM images showing individual nanoparticles following extensive dialysis. (B) Size distribution obtained from measuring 85 individual particles.

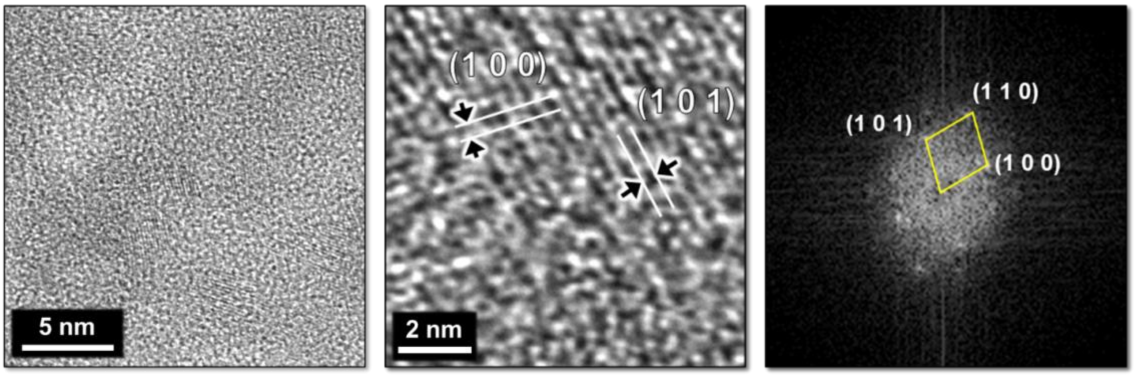

Fig. S3. High-resolution TEM images of **1**. The left image clearly showing the crystalline nature of the material. The center image shows the atomic arrangement, showing interplanar distances of 2.41 Å and 2.12 Å, corresponding to the (1 0 0) and (1 0 1) Miller indices, respectively. At right is a fast Fourier transform (FFT) of the center image, additionally revealing the periodicity along the (1 1 0) direction.

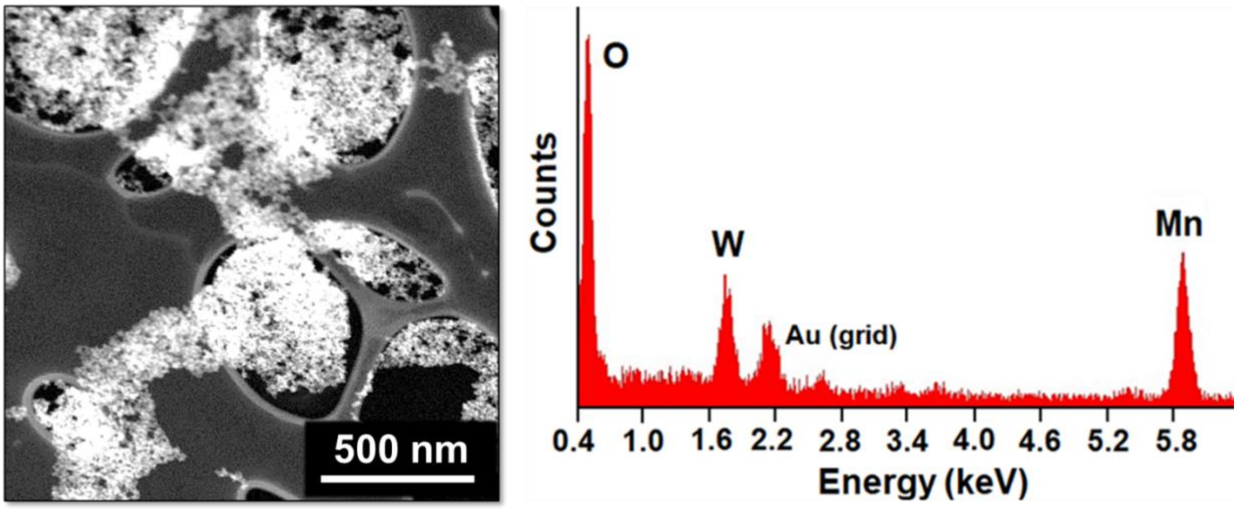

Fig. S4. EDX spectrum of **1**. Left is the scanning transmission electron microscopy (STEM) image of the selected area for EDX mapping analysis shown in Figure 2C. The homogeneous distribution of the Mn, O and W can be seen in the EDX spectrum on the right. (B) EDX spectrum of (A), showing the peaks for O, Mn and W. The elemental distribution is found as 69.9 : 24.1 : 5.88 (percent) for O : Mn : W. A small peak stemming from the Au TEM grid used is also observed around 2.1 keV.

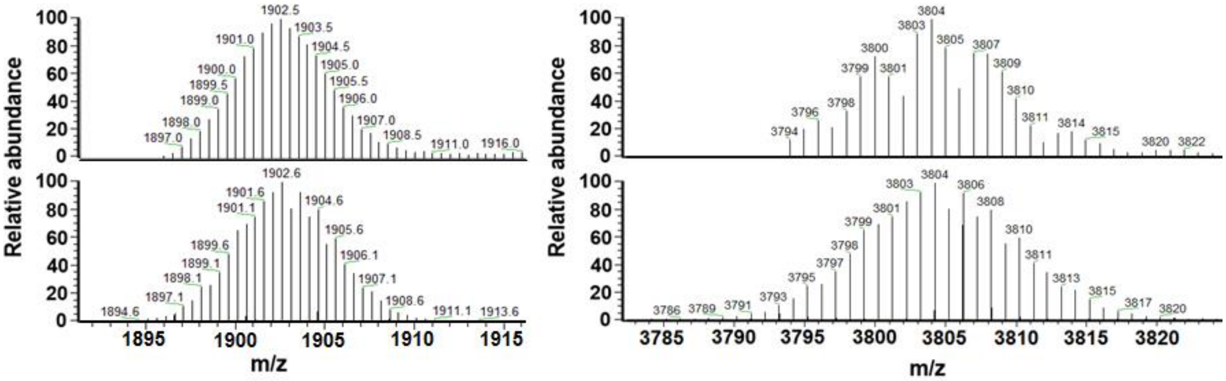

Fig. S5. ESI-MS spectra of the POM capping ligands, both the experimental (top) and simulated (bottom) are shown. The left spectrum corresponds to m/z = 2 for the species $KHMnTBA_4[AlCr(V)W_{11}O_{40}]^{2+}$, the right spectrum corresponds to m/z = 1 for the species $KMnTBA_4[AlCr(V)W_{11}O_{40}]^{+}$.

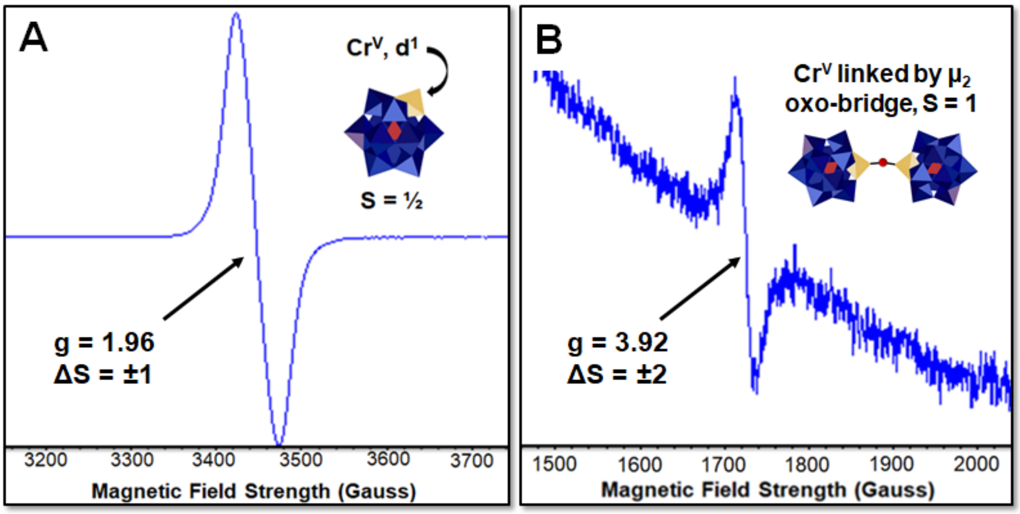

Fig. S6. Electron paramagnetic resonance spectra of the reaction byproduct $[AlCr^VW_{11}O_{40}]^{6-}$. (A) A single transition found at g = 1.96 corresponding to a ΔS = ±1, attributed to the d^1 Cr^V atom found in the POM. (B) A single, formally forbidden, transition found at g = 3.92 corresponding to a ΔS = ±2, attributed to the dimeric species $[(AlCr^VW_{11}O_{39})_2\text{-}(\mu_2\text{-}O)]^{10-}$.

Table S1. POM coverage on the MnO_2 cores of 1.

Calculation of POM coverage of **1**, considering a 2.7 nm (Diameter) ideal spherical ε- MnO_2 NC core and 1.8 nm^2 POM footprint on the surface. Atomic fractions of Mn : W were obtained from ICP-OES.

Density of MnO_2	$5.03\cdot10^{-21}$ g·nm^{-3}
M.W. of MnO_2	86.9368 g·mol^{-1}
Volume of MnO_2 sphere	10.30 nm^2
**Mn atoms per particle**	**359**
Surface area of MnO_2 sphere	22.89 nm^2
POM footprint	1.8 nm^2
POM/particle (max. based on footprint)	13
**Atom/atom fraction of Mn=**	**0.83**
**Atom/atom fraction of W=**	**0.17**
**POM/particle**	**7.7**
Total number of W + Mn atoms per complex	443

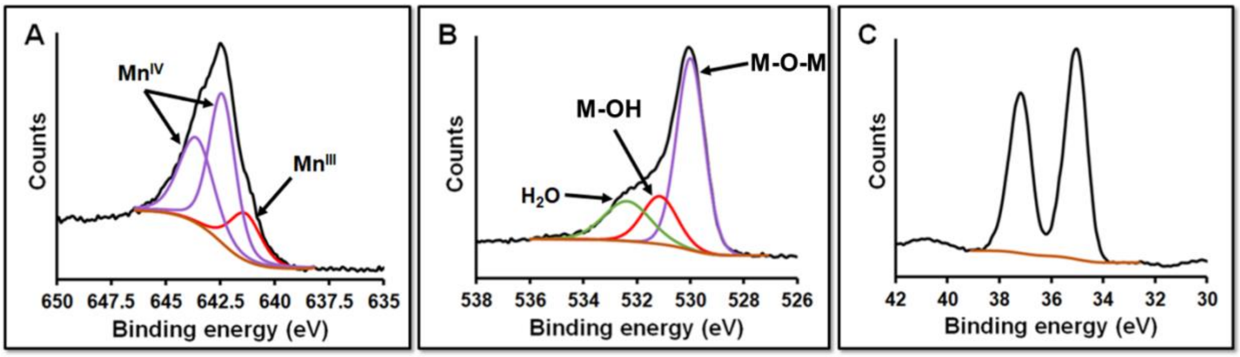

Fig. S7. High-resolution XPS spectra of **1.** (A) Deconvoluted spectrum of the Mn2*p* 3/2 transition, indicating two groups of binding energies, 643.3 and 642.2 eV corresponding to Mn(IV), and 641.0 eV corresponding to Mn(III). (B) Deconvoluted spectrum of the O1*s* transition, indicating three groups of binding energies, 529.9 eV corresponding to M-O-M oxo groups (M = W, Mn), 531.1 eV corresponding to M-OH hydroxyl groups, and 532.3 eV corresponding to adsorbed water. (C) Spectrum of the W4*f* transition, indicating the presence of W^{VI} in the complex due to the presence of the POM-ligands in **1**.

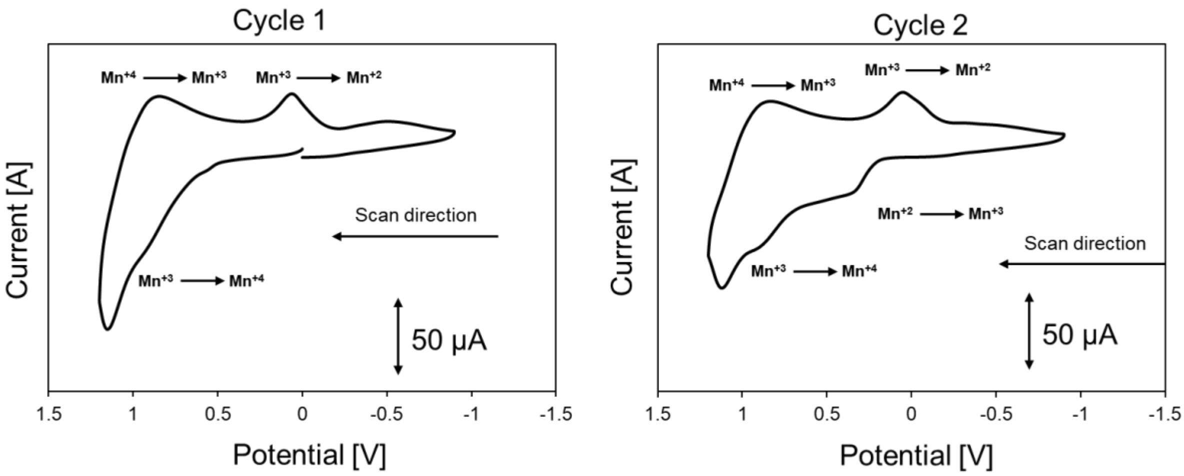

Fig. S8. Cyclic voltammograms of **1**. Left: first cycle, beginning at 0 V bias, scanning towards to positive direction, shows the first oxidation of Mn(III) to Mn(IV) at around 0.9 V. Going in the reverse direction shows two cathodic peaks, reduction of Mn(IV) to Mn(III) around 0.85 V, and reduction of Mn(III) to Mn(II) around -0.05 V. Right: subsequent cycle now shows, in addition to the previous peaks, the oxidation of Mn(II) to Mn(III) around 0.2 V.

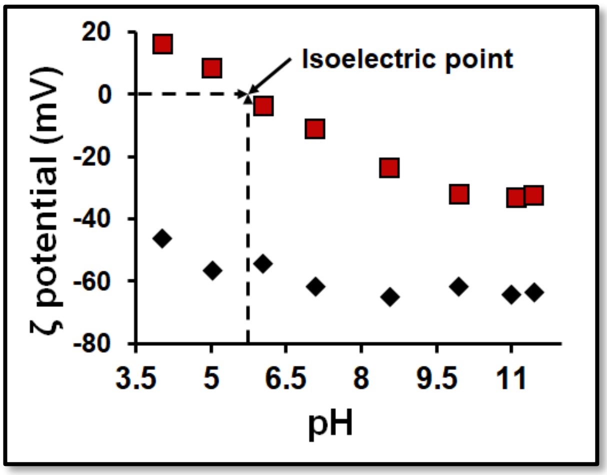

Fig. S9. Zeta potential measurement over a wide pH range (4-11.5), showing that the overall surface charge of **1** (black diamonds) is highly negative, diagnostic for anionic stabilization of the ε-MnO_2 NCs by the POM-ligands. The zeta potential measurement for colloidal MnO_2 (red squares) is shown for comparison, lacking stability due to lack of protecting ligands. The native pH of the proton form of **1** is 5.

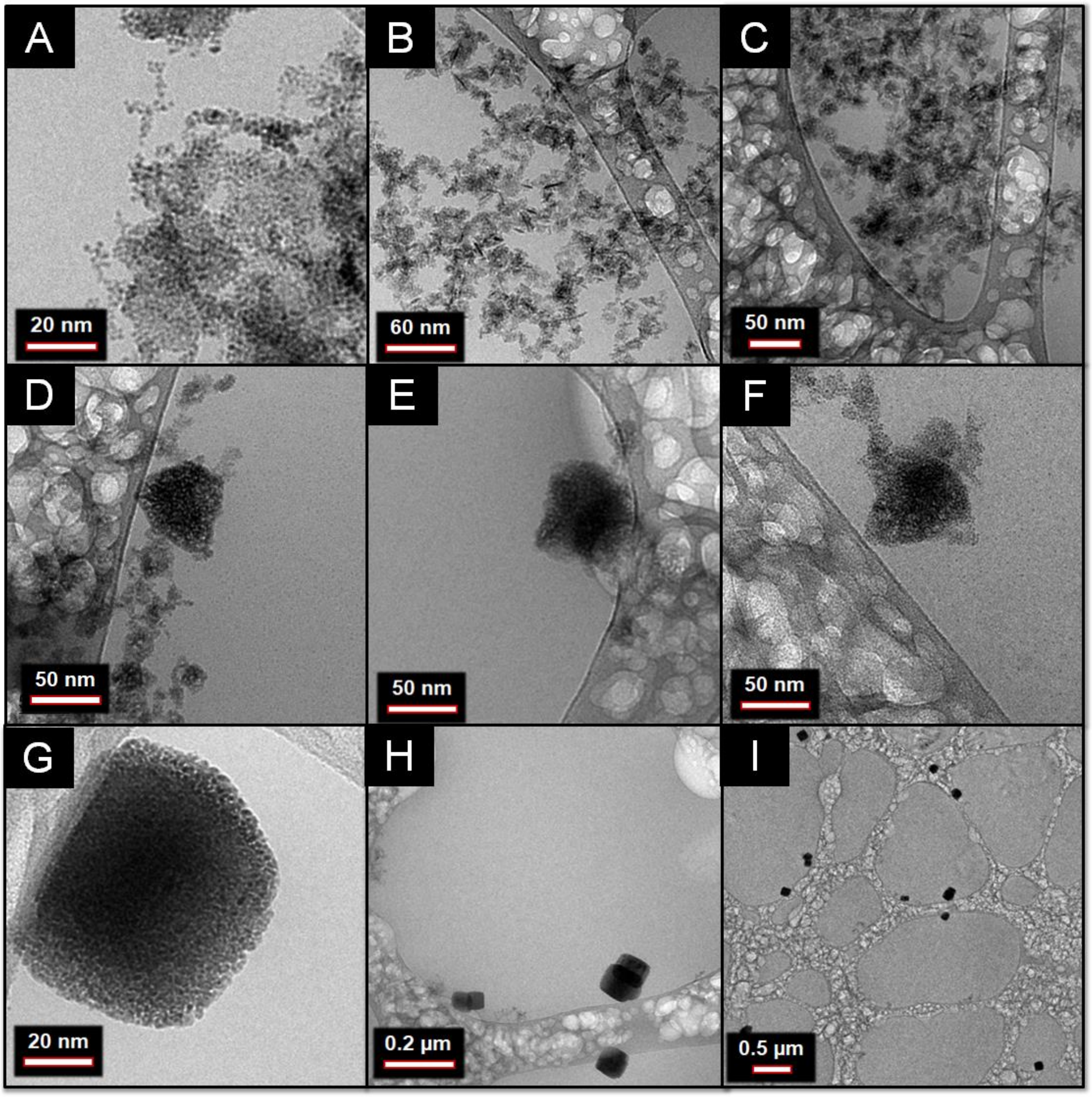

Fig. S10. Cryo-TEM images of solutions containing **1** and varying amounts of K^+ ions per POM (from ICP-OES). (A-C) Images of a solution containing 8.8 eq K^+ to POM, showing undefined aggregations of individual particles of **1**. (D-F) Images of a solution containing 13.2 eq K^+ to POM, showing more well formed assemblies consisting of numerous particles of **1**. (G-I) Images of a solution containing 17.6 eq K^+ to POM, well formed cubic assemblies consisting of numerous particles of **1**, up to 28,000 individual complexes. In particular, Figures G and I showing numerous such assemblies, with Figure G being a magnification of the central region in panel I.

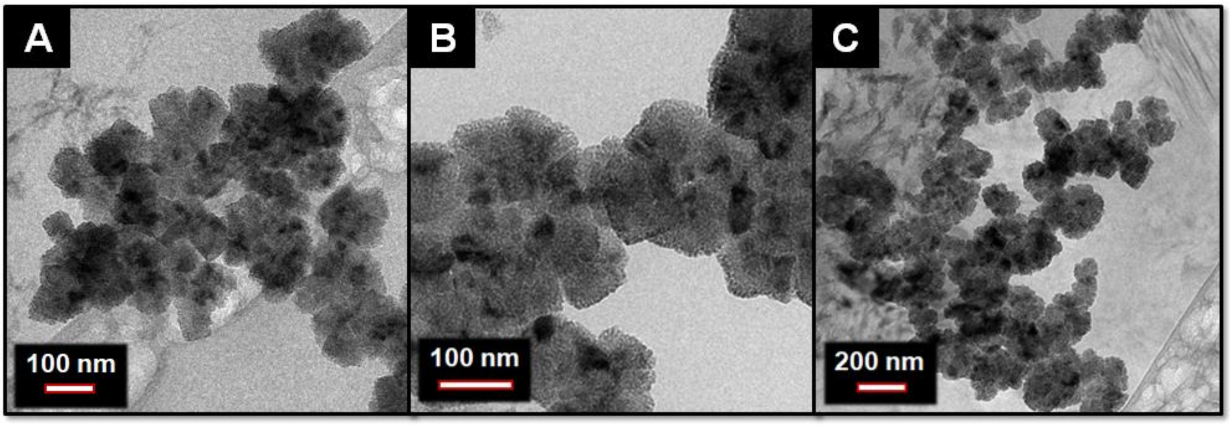

Fig. S11. (A-C) Cryo-TEM images of a solution containing 650 eq. K^+ to POM ($[K^+]$ = 10 mM), showing aggregations consisting of the cubic assemblies, composed from millions of individual complexes of **1**.

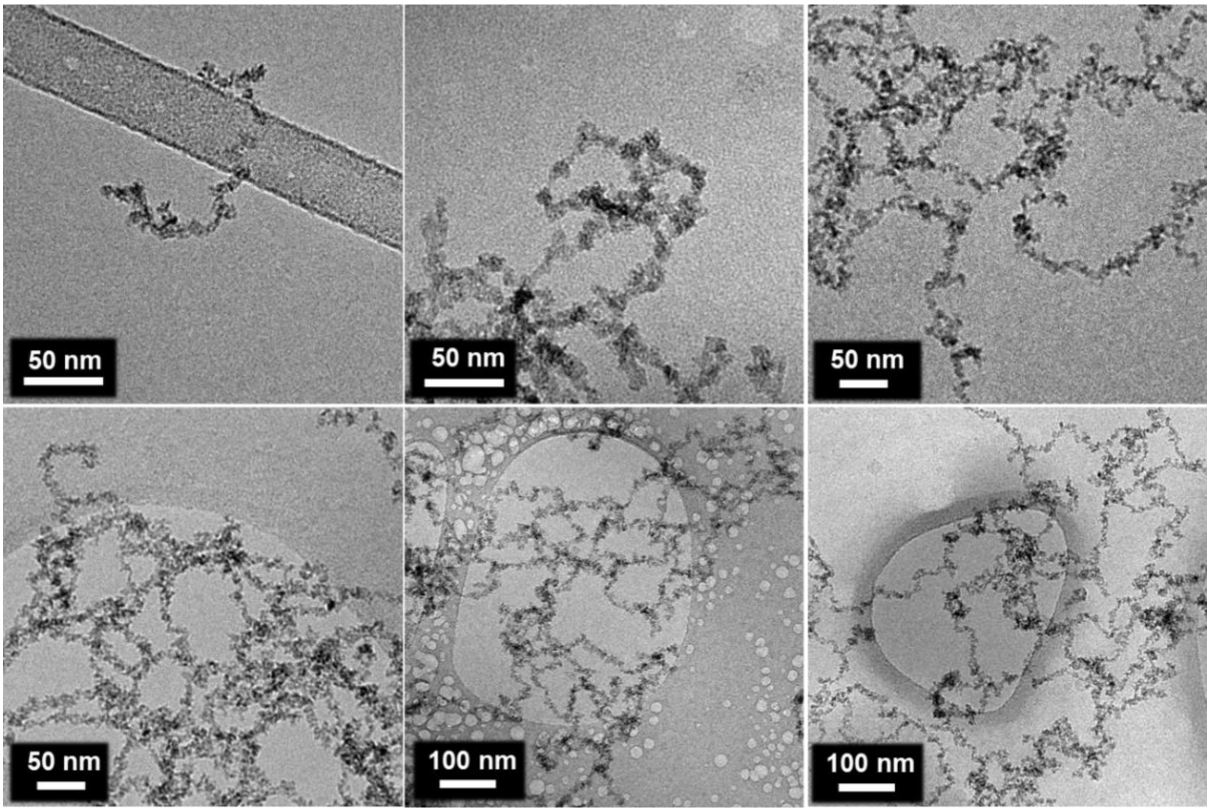

Fig. S12. Cryo-TEM images of a solution containing **1**, seconds after addition of 17.6 K^+ per POM, showing the formation of fractal aggregates.

Fractal analysis. The following procedure is based on the standard grid (box-counting) method as discussed by Klinkenberg.[13] The box counting analysis was done in Python, using NumPy/SciPy for the mask and counting routines.

First, we convert the grayscale image $I(x, y)$ to a binary mask $B(x, y)$ by selecting "very-dark" pixels relative to the local background using:

$$I_{th}(x,y) = \underbrace{\frac{1}{(2w+1)^2} \sum_{u=-w}^{w} \sum_{v=-w}^{w} I(x+u, y+v)}_{local\ mean\ over\ window\ size\ (2w+1)} - \Delta$$

$$B(x,y) = \begin{cases} 1, & I(x,y) < I_{th}(x,y) \\ 0, & otherwise \end{cases}$$

Here we used $w = 50$, i.e. a 101x101 px window, and an empirical constant intensity offset that we subtract from the local mean, $\Delta = 40$, to include genuine branches while excluding the carbon film of the TEM grid, yielding:

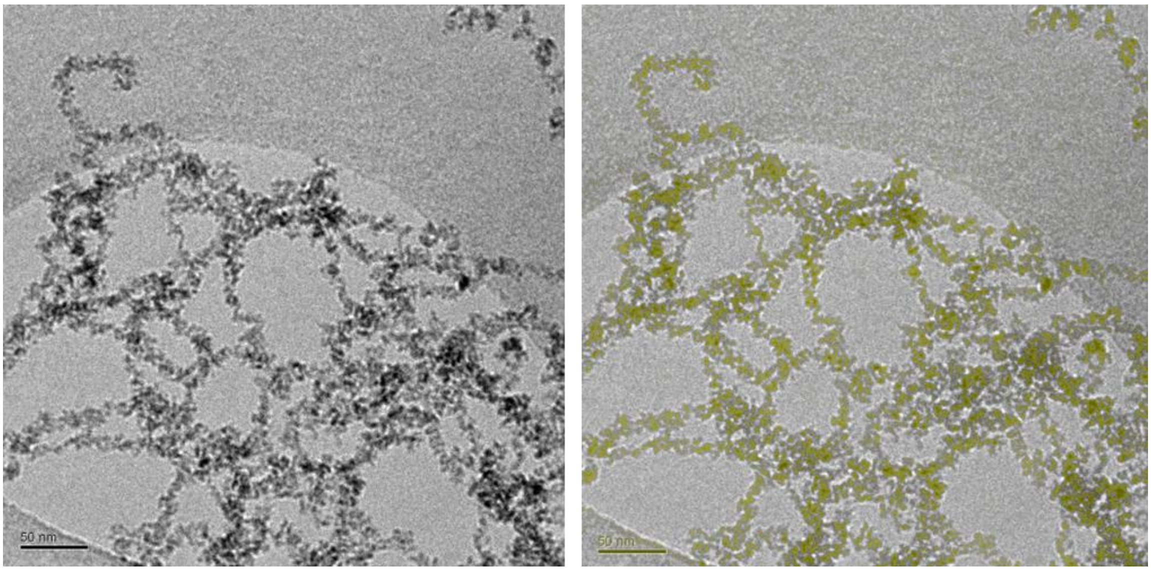

Fig. S13. (Left) Grayscale cryo-TEM micrograph of an aggregate region 15 seconds after addition of K^{+}. (Right) The same field with the binary mask overlaid in semi-transparent yellow (Δ=40, local mean over 101-pixel window minus 40 intensity units), highlighting the pixels used for box-counting in the fractal dimension analysis.

Next, we define box sizes: let $L_0 = 1024$ be the original image width and height in pixels, we choose box edge lengths as:

$$\varepsilon_k = 2^k, \quad k = \log_2(L_0), \log_2(L_0 - 1), \dots, 1$$

This ensures we find the grids in which the grid tiles the image exactly with no partial boxes (basically, grids such as 1024 x 1024 with 1 tile, 512 x 512 with 4 tiles, 256 x 256 with 16 tiles, etc.).

Next, we count *occupied* boxes: for each ε_k, we overlay a non-overlapping grid of $\varepsilon_k \times \varepsilon_k$ boxes. Define:

$$b_{ij}^{(k)} = \begin{cases} 1, & \sum_{x=i\varepsilon_k}^{(i+1)\varepsilon_k-1} \sum_{y=j\varepsilon_k}^{(j+1)\varepsilon_k-1} B(x,y) > 0 \\ 0, & otherwise \end{cases}$$

And the total count of occupied boxes:

$$N(\varepsilon_k) = \sum_{i,j} b_{ij}^{(k)}$$

For example:

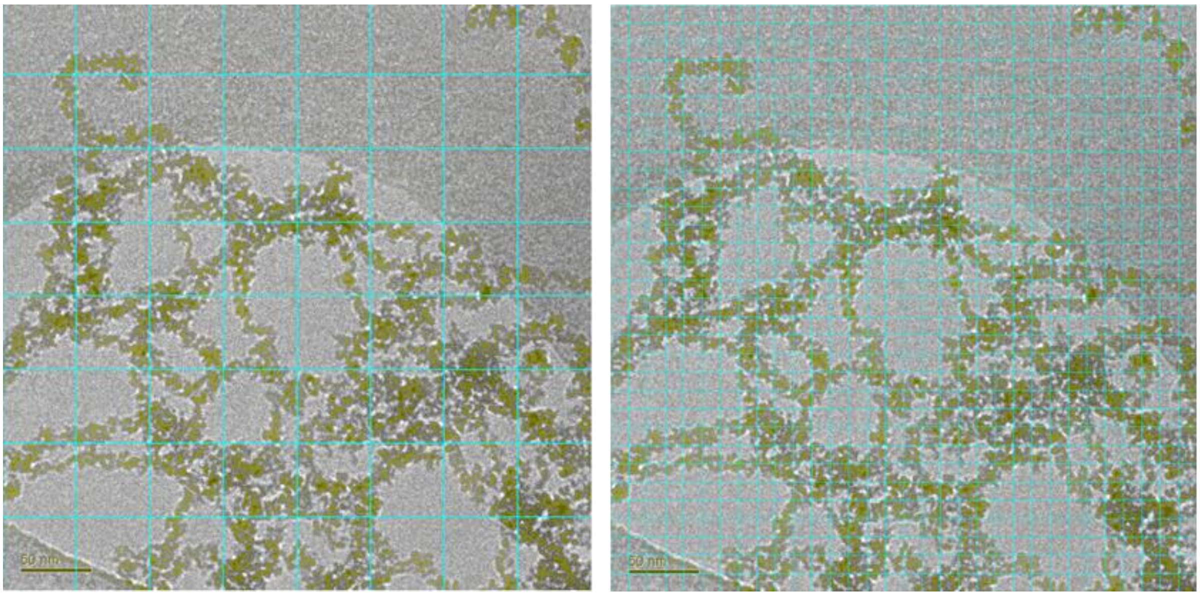

Fig. S14. Box-counting grids and mask overlays for the masked image. Left - 128-pixels grid. Right - 32-pixels box grid.

In addition, to remove possible residual noise from the masking procedure, we remove all components with an area of 20 pixels. Also, we limit the analysis to box sizes no smaller than 16-pixels because at finer scales pixelation and residual thresholding noise dominate, breaking the true fractal power-law behavior.

Finally, we use the power-law scaling to find *D*, the fractal dimension:

$$N(\varepsilon) \propto \varepsilon^{-D} \quad \longrightarrow \quad \ln\big(N(\varepsilon_k)\big) = -D\ln(\varepsilon_k) + C$$

Fitting a straight line to the $\{\log(\varepsilon_k)\ ,\log\big(N(\varepsilon_k)\big)\}$ data, and then fitting a least squares linear fit, yields:

$$slope = -D, \quad D = -\frac{d\ln\big(N(\varepsilon_k)\big)}{d\ \ln(\varepsilon_k)}$$

The following table contains the results of the analysis:

Table S2. Box-counting results for aggregates with natural logarithms of box size and counts.

Box size (px)	Total cells	Cells w/ mask pixels	ln (size)	ln (count)
1024	1	1	6.931472	0
512	4	4	6.238325	1.3862944
256	16	12	5.545177	2.4849066
128	64	47	4.85203	3.8501476
64	256	167	4.158883	5.1179938
32	1024	544	3.465736	6.2989492
16	4096	1464	2.772589	7.2889277

The resulting plot from which $D = -\frac{d\ln(N(\varepsilon_k))}{d\ \ln(\varepsilon_k)}$ was extracted:

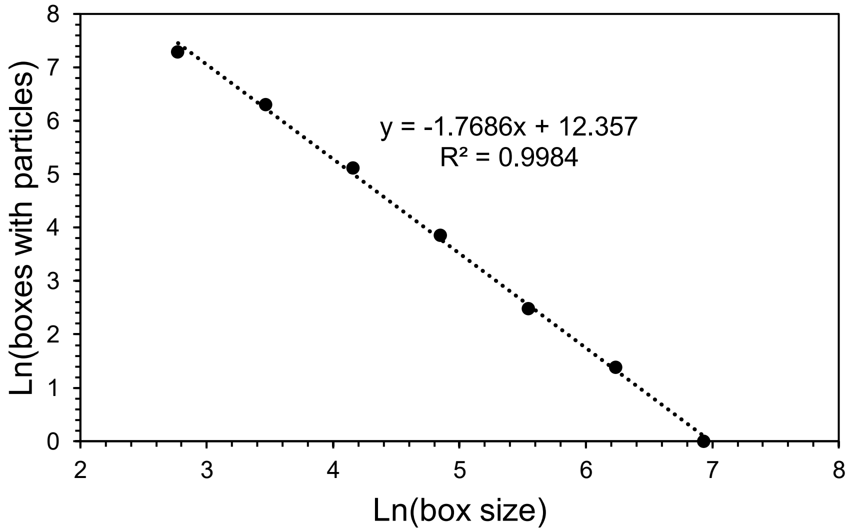

Fig. S15. Linear regression of the ln(amount of boxes with particles) vs ln(box size) yields the fractal parameter, *D*, of the particles in the image, with *D* = 1.77.

Thus giving a *D* = 1.77 as the fractal value for the particle arrangement in the image.

Analyzing a range of images in the above manner yields an average fractal parameter, *D* = 1.75 ± 0.04.

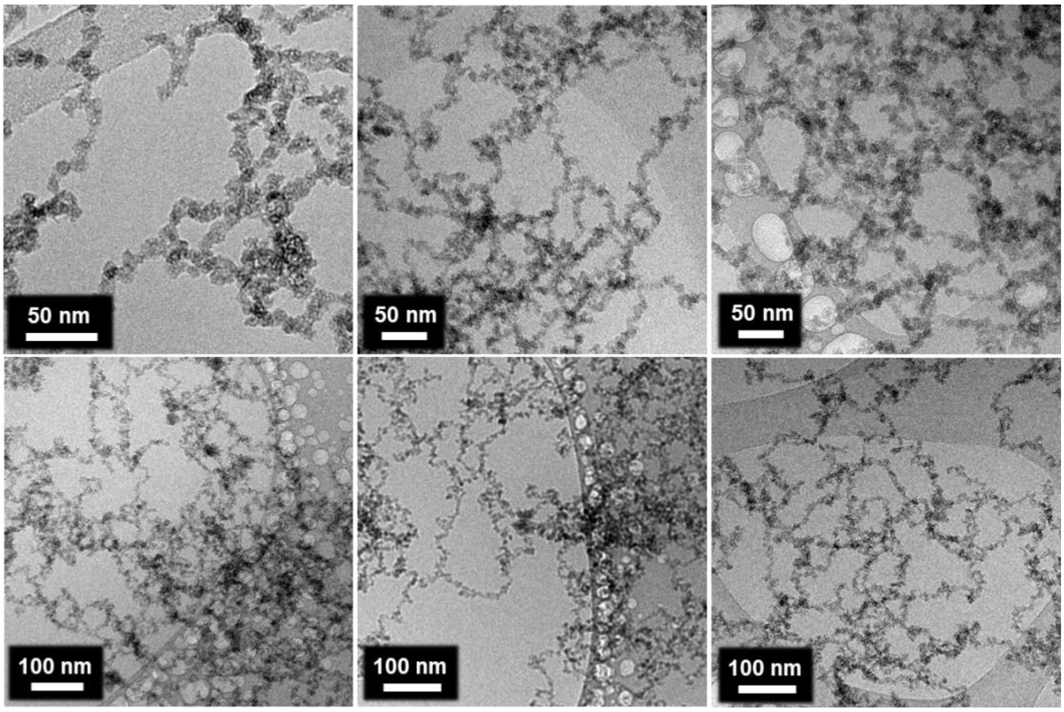

Fig. S16. Cryo-TEM images of a solution containing **1**, 5 min after addition of 17.6 K^+ per POM, showing the initial aggregation of fractal aggregates.

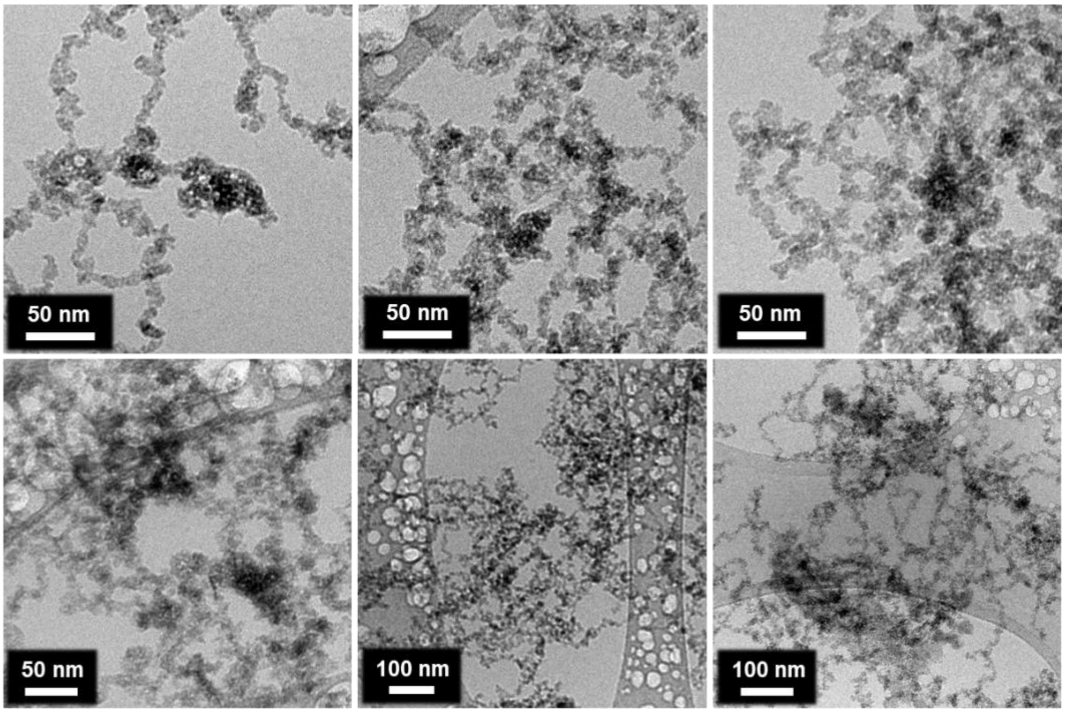

Fig. S17. Cryo-TEM images of a solution containing **1**, 20 min after addition of 17.6 K^+ per POM, showing aggregation of at the branching nodes of fractal aggregates and formation of dense nodes.

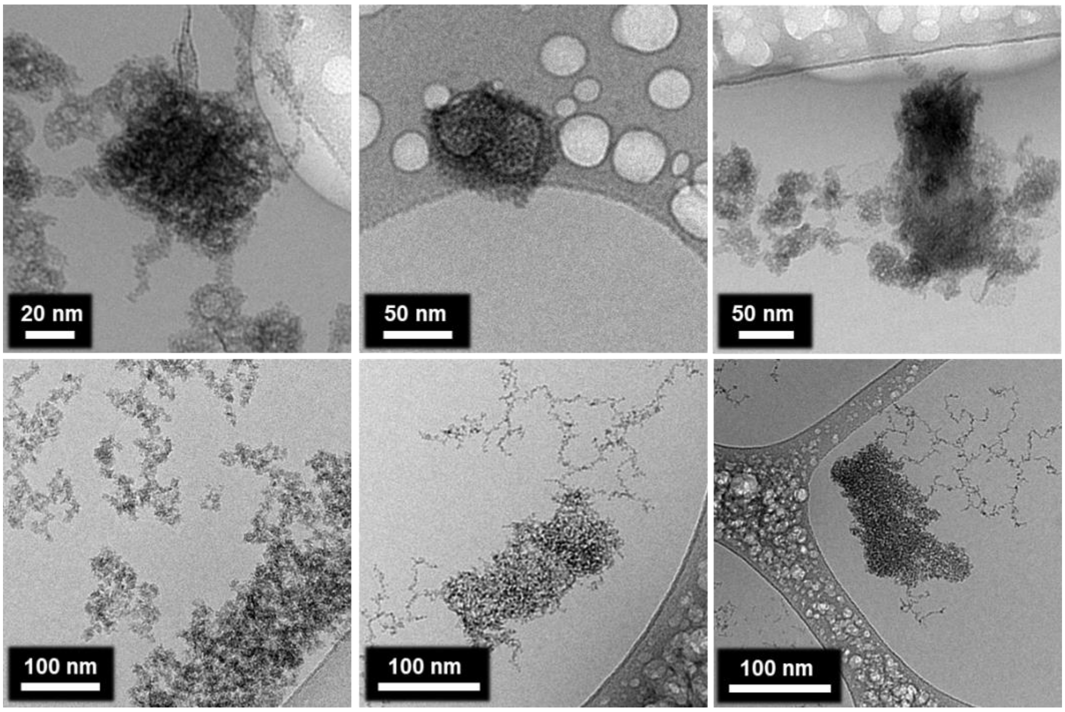

Fig. S18. Cryo-TEM images of a solution containing **1**, 1 h after addition of 17.6 K^+ per POM, showing large diffuse clusters at what were previously branching nodes, with marked depletion of the previously present fractal aggregate strands.

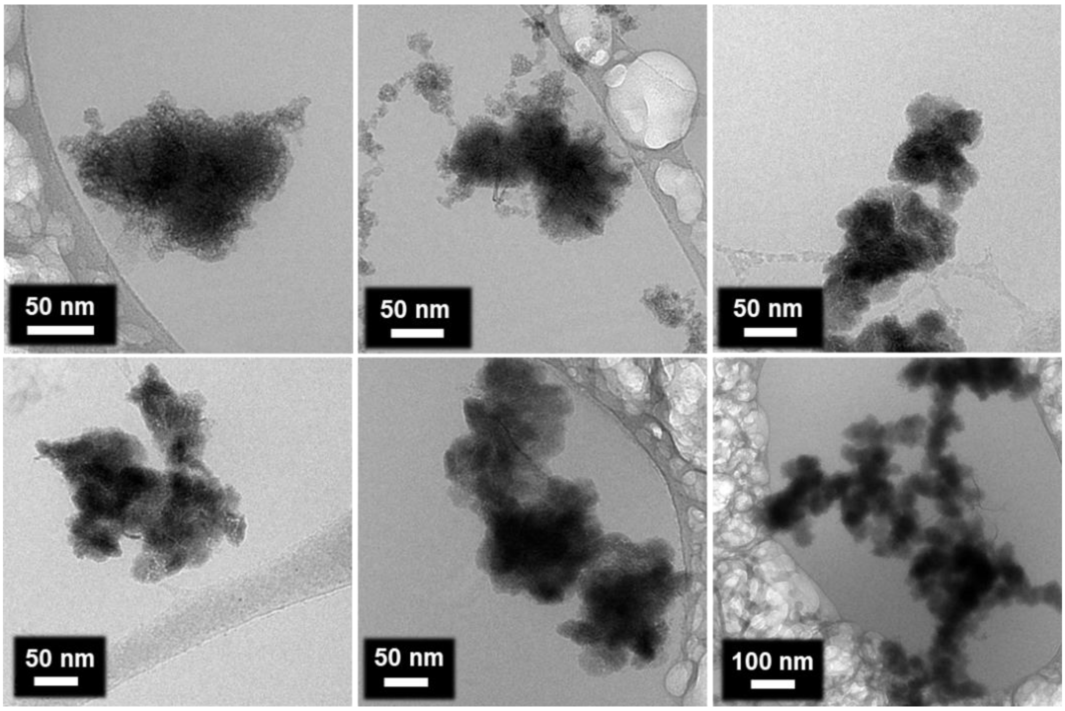

Fig. S19. Cryo-TEM images of a solution containing **1**, 5 h after addition of 17.6 K^+ per POM, with highly dense 3D structures, evident from the stronger contrast compared to Figure S15, suggesting that several clusters have joined together.

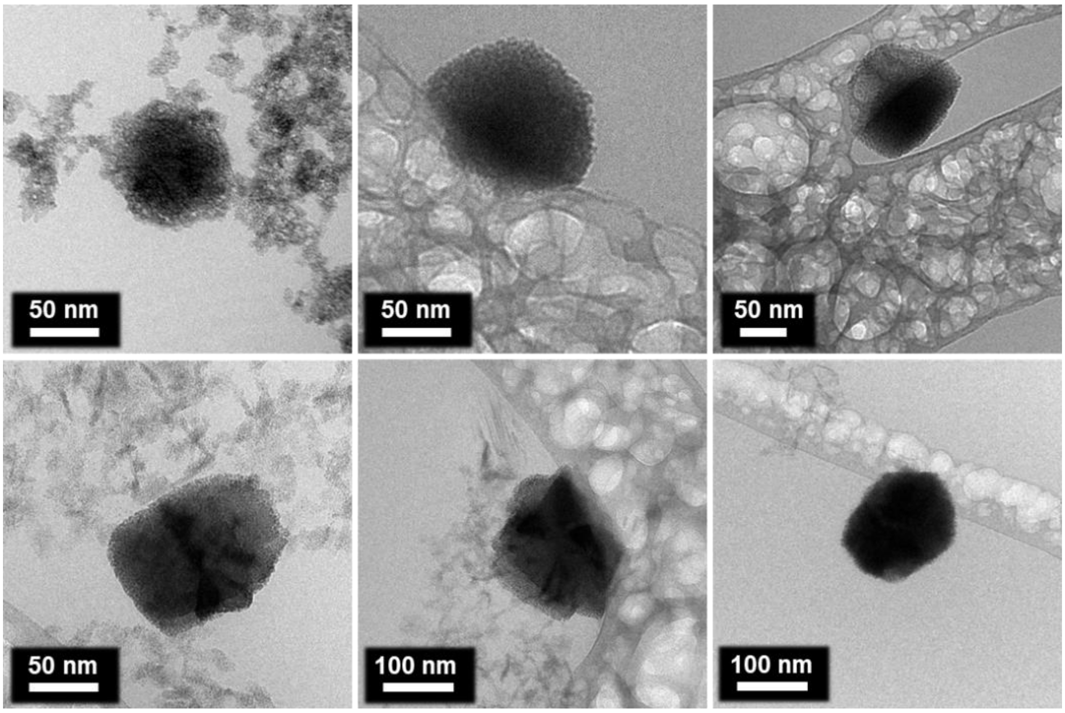

Fig. S20. Cryo-TEM images of a solution containing **1**, 24 h after addition of 17.6 K^+ per POM, showing various degrees of crystallization of the dense 3D structures seen in Figure S16.

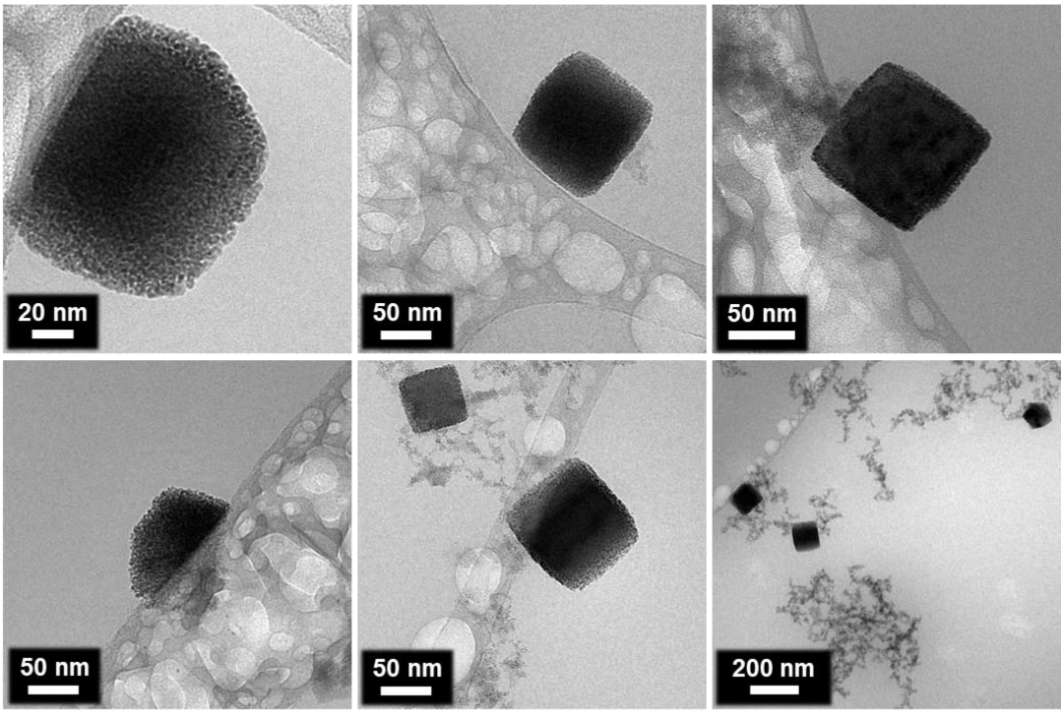

Fig. S21. Cryo-TEM images of a solution containing **1**, 7 days after addition of 17.6 K^+ per POM, showing well formed cubic structures. It is interesting to note that some fractal aggregates are still visible.

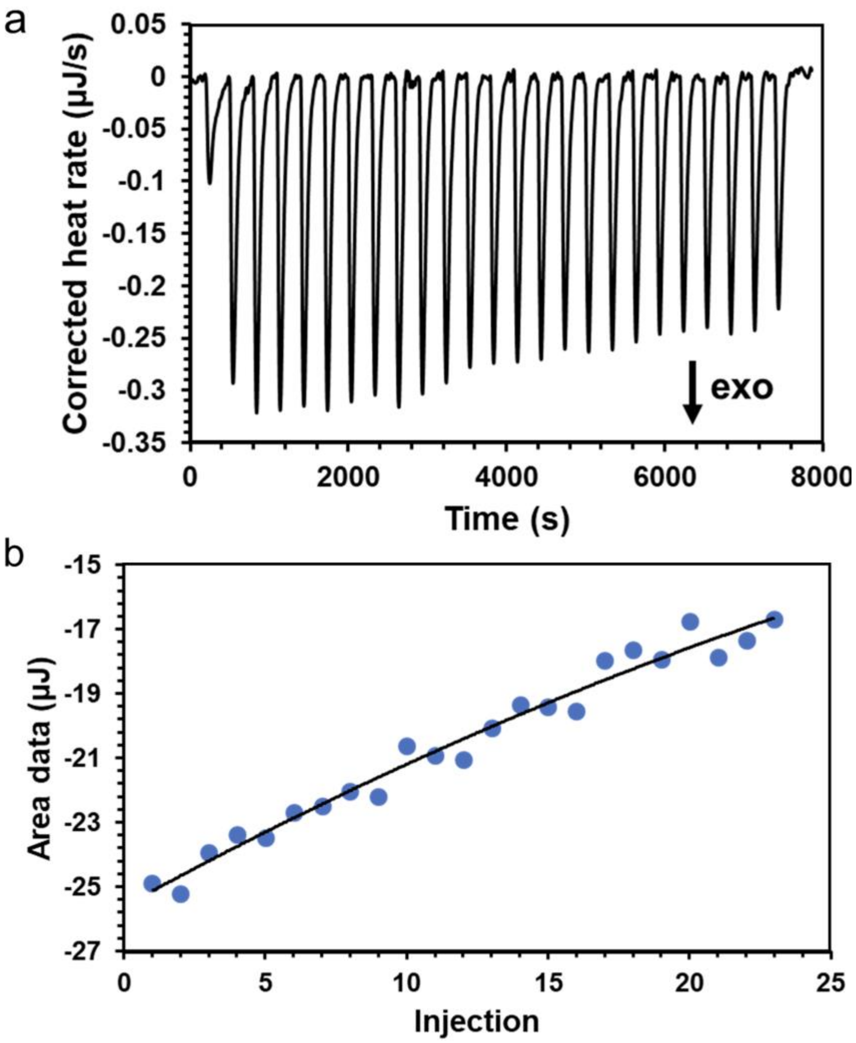

Fig. S22. Isothermal titration calorimetry of branching-node formation and growth. (a) Corrected heat rates during additions of 5 μl aliquots of K^+ (4.4 mM) over 4 hours into a 1 ml solution of **1** (3.34 μM), to a final ratio 17.6 K^+ ions per POM ligand. (b) Integrated areas of each peak in (a), represented by the blue dots, were fitted to an independent site model[14] (black curve). The first peak was used for calibration and is not included in modeling.

Table S3. Thermodynamic parameters for titration of K^+ into aqueous 1[a]

K **(mM)**	**Δ*H*** **(kcal·mol^{-1})**	**Δ*S*** **(J·mol^{-1}·K^{-1})**	**-TΔ*S*** **(kcal·mol^{-1})**	**Δ*G*** **(kcal·mol^{-1})**
3.7 ± 0.3	-33.6 ± 3	-424.7	30.2	-3.4

[a]Parameters were obtained using an independent-site model.[14]

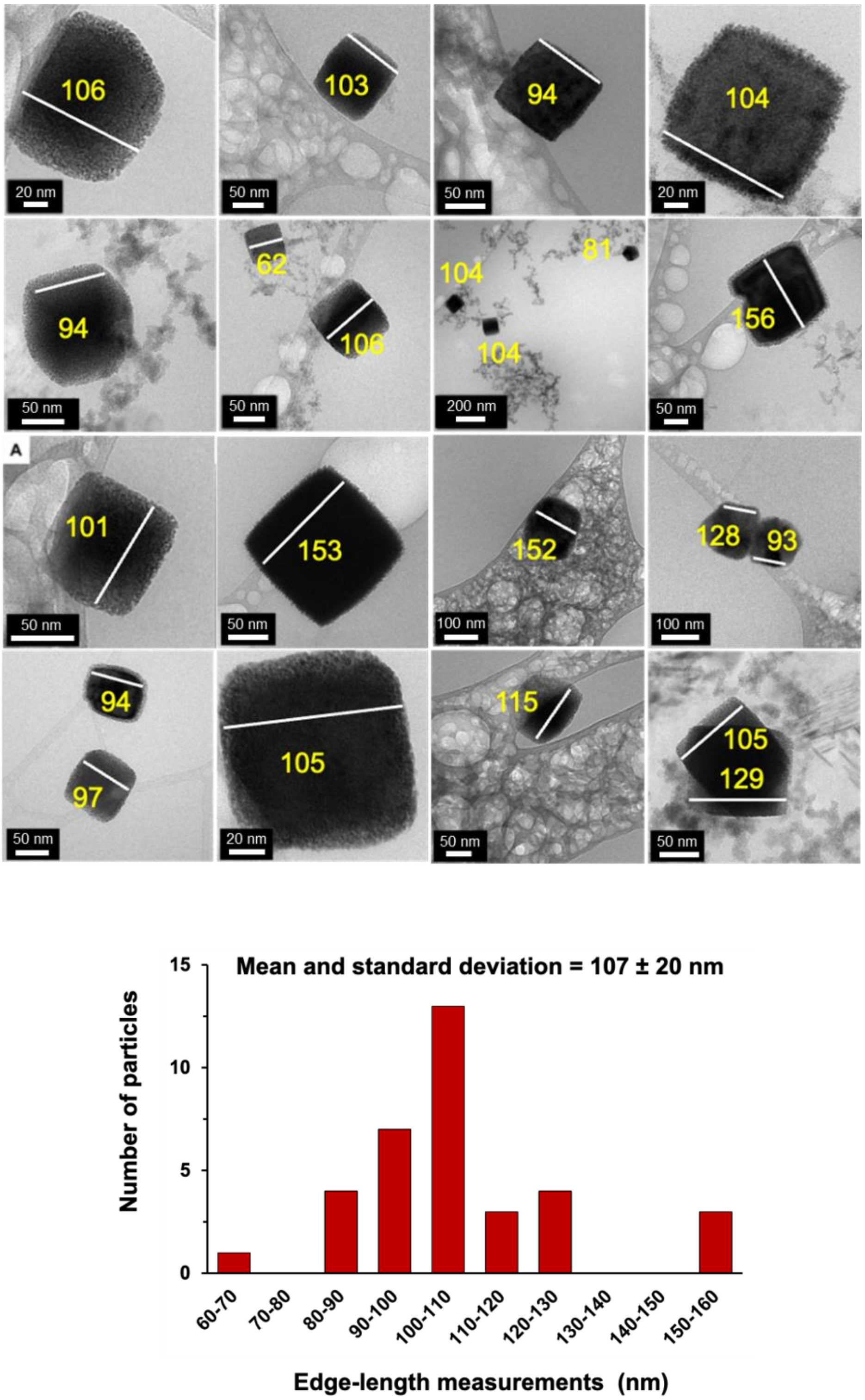

Fig. S23. Top: Cryo-TEM images of soluble lattices of **1**, showing examples of measurement along their edges. Bottom: Size distribution obtained from measuring 35 crystallites.

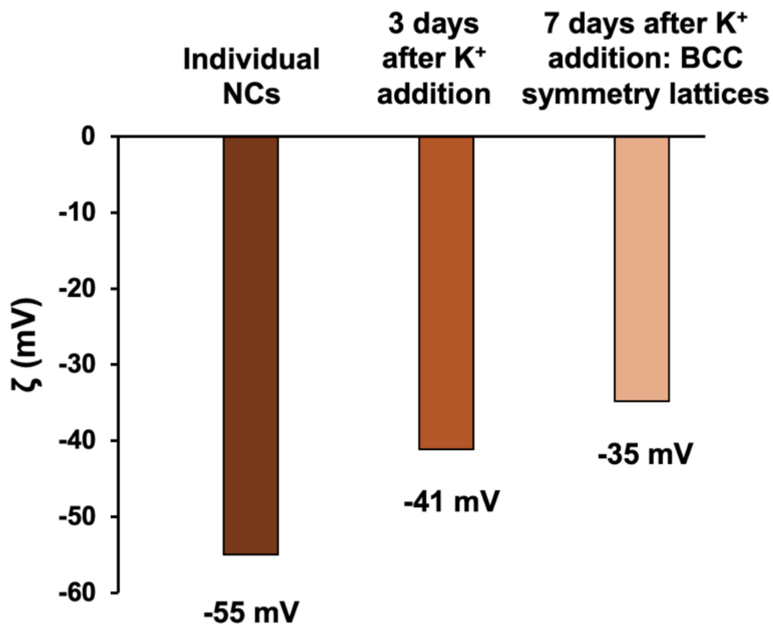

Fig. S24. Zeta potential values, in mV, at various intervals after addition of K^+.

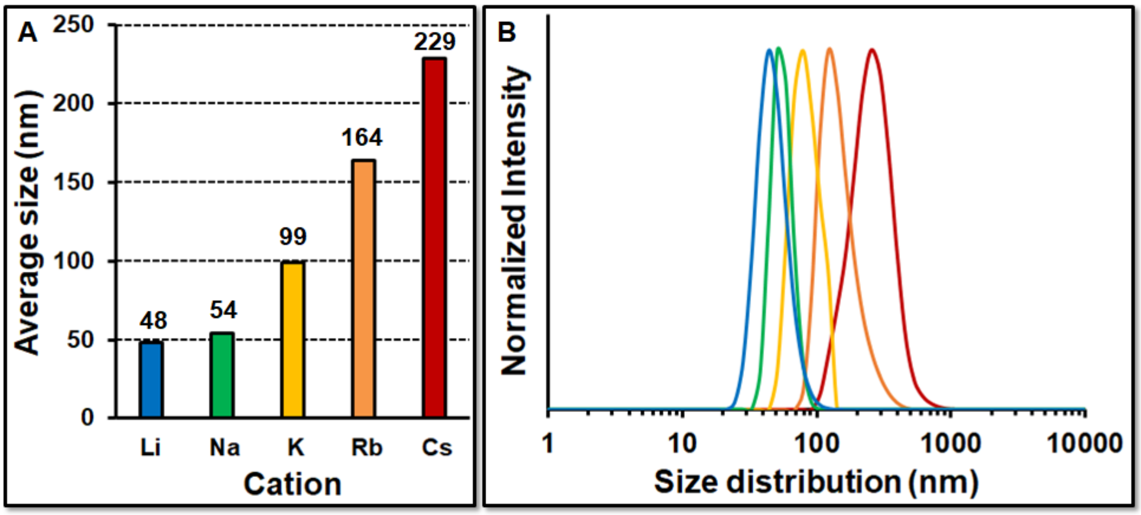

Fig. S25. Ion-pairing interaction experiments between the POM-ligands on the surface of **1** and alkali metal cation at a constant concentration of 5 mM per cation. Color code: Cs – red, Rb – orange, K – yellow, Na – green, Li – blue. (**A**) Average diameter values obtained from number weighted DLS measurements showing an increase in particle size present in the solutions. (**B**) DLS spectra showing the size distribution of the various solutions.

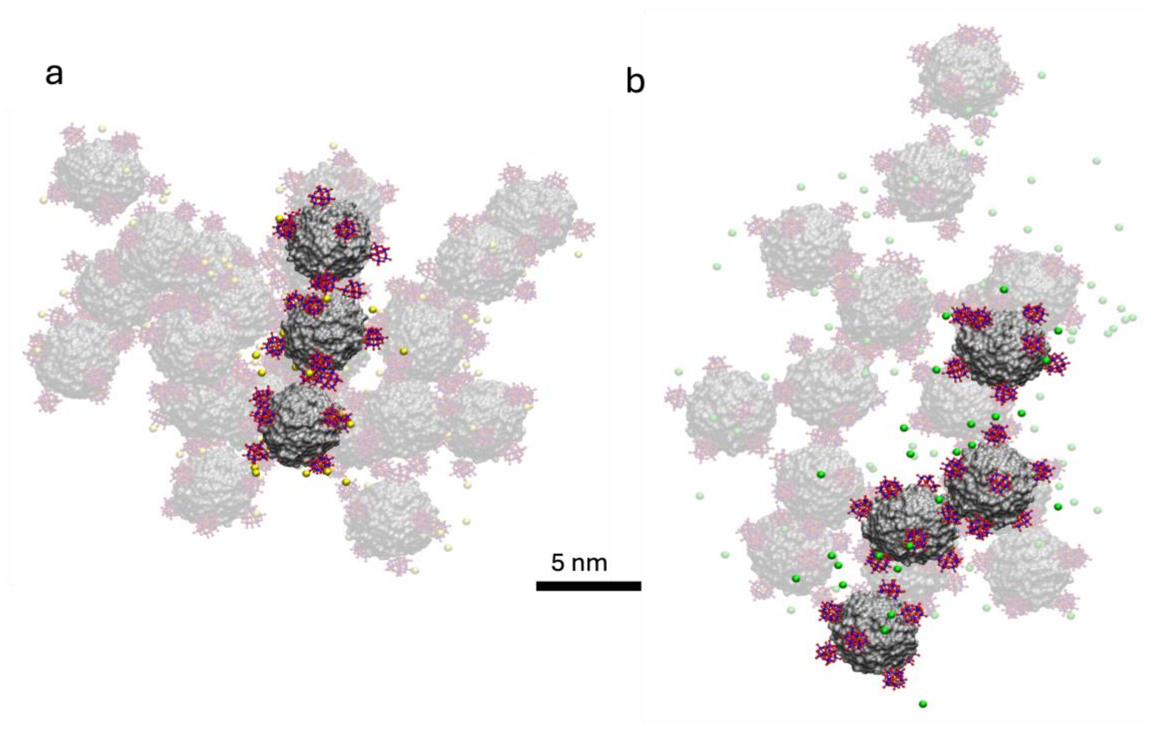

Fig. S26. (a) POM-NPs self-assembly in the presence of Li^+ cations. **(b)** The same in the presence of K^+ cations. The system of 32 NPs, initially at center-to-center distances of 10 nm, and 224 K^+ or Li^+ anions was simulated in $15.6{\times}10^3$ nm^3 water box; obtained in 100 ns simulations done in NpT ensemble at T = 300 K and P = 1 bar. Gray, magenta, yellow, and green colors represent ε-MnO_2 core, POM ligands, Li^+ and K^+ ions, respectively. The data show that early on in assembly, POMs ligands interact with the positively charged (partially protonated) surfaces of neighboring NCs.

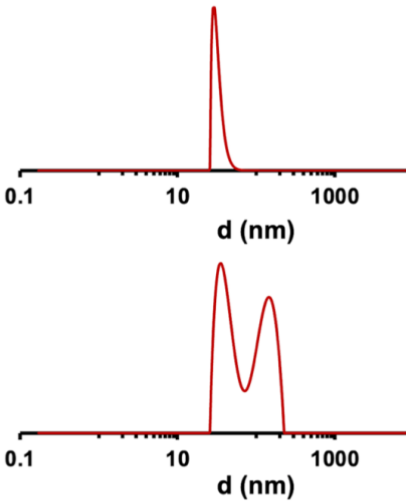

Fig. S27. Number weighted (top) and unweighted (bottom) DLS data for a solution of **1** one week after adding 17.6 equiv. Li^+ per POM ligand.

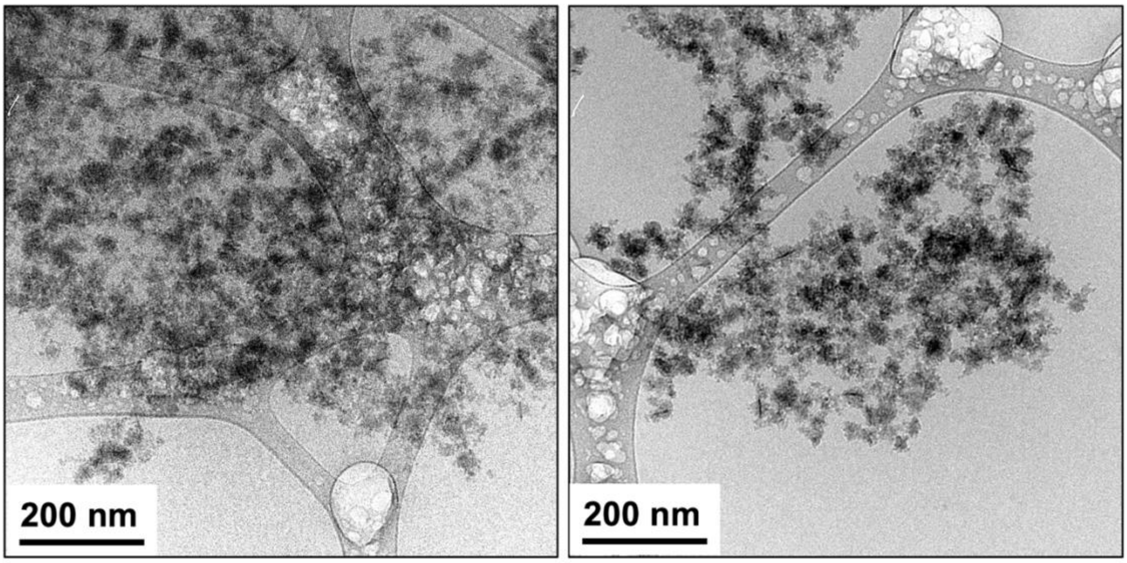

Fig. S28. Cryo-TEM images of a solution of **1** one week after adding 17.6 equiv. Li^+ per POM ligand.

References

(1) Cowan, J. J.; Bailey, A. J.; Heintz, R. A.; Do, B. T.; Hardcastle, K. I.; Hill, C. L.; Weinstock, I. A. Formation, isomerization, and derivatization of keggin tungstoaluminates. *Inorg. Chem.* **2001**, *40*, 6666-6675.

(2) Perez-Benito, J. F.; Brillas, E.; Pouplana, R. Identification of a soluble form of colloidal manganese(IV). *Inorg. Chem.* **1989**, *28*, 390-392.

(3) Rong, C.; Anson, F. C. Simplified preparations and electrochemical behavior of two chromium-substituted heteropolytungstate anions. *Inorg. Chem.* **1994**, *33*, 1064-1070.

(4) Bielański, A.; Lubańska, A. FTIR investigation on Wells–Dawson and Keggin type heteropolyacids: dehydration and ethanol sorption. *J. Mol. Cat. A* **2004**, *224*, 179-187.

(5) Goodgame, D. M. L.; Joy, A. M. EPR study of the Cr(V) and radical species produced in the reduction of Cr(VI) by ascorbate. *Inorg. Chim. Acta* **1987**, *135.*, 115-118.

(6) Lahootun, V.; Karcher, J.; Courillon, C.; Launay, F.; Mijares, K.; Maatta, E.; Proust, A. A (nitrido)chromium(V) function incorporated in a Keggin-type polyoxometalate: $[PW_{11}O_{39}CrN]^{5-}$ – Synthesis, characterization and elements of reactivity. *Eur. J. Inorg. Chem.* **2008**, *2008*, 4899-4905.

(7) Khenkin, A. M.; Hill, C. L. Oxo transfer from high-valent totally inorganic oxometalloporphyrin analogs, $[X^{n+}W_{11}O_{39}CrVO]^{(9-n)-}$ ($X^{n+} = P^{5+}$, Si^{4+}), to hydrocarbons. *J. Am. Chem. Soc.* **1993**, *115*, 8178-8186.

(8) Maurya, B. P.; Ikram, M.; Khan, S.; Singh, R. J. S = 12, S = 1 and S = 2 EPR spectra in copper doped $KHSO_4$ single crystal. *Solid State Commun.* **1996**, *98*, 843-845.

(9) Richert, S.; Tait, C. E.; Timmel, C. R. Delocalisation of photoexcited triplet states probed by transient EPR and hyperfine spectroscopy. *J. Mag. Res.* **2017**, *280*, 103-116.

(10) Antonio, M. R.; Nyman, M.; Anderson, T. M. Direct observation of contact ion-pair formation in aqueous solution. *Angew. Chem. Int. Ed.* **2009**, *121*, 6252-6256.

(11) Kistler, M. L.; Bhatt, A.; Liu, G.; Casa, D.; Liu, T. A complete macroion−"blackberry" assembly−macroion transition with continuously adjustable assembly sizes in {Mo132} water/acetone systems. *J. Am. Chem. Soc.* **2007**, *129*, 6453-6460.

(12) Uchida, S. Frontiers and progress in cation-uptake and exchange chemistry of polyoxometalate-based compounds. *Chem. Sci.* **2019**, *10*, 7670-7679.

(13) Klinkenberg, B. A review of methods used to determine the fractal dimension of linear features. *Math. Geol.* **1994**, *26*, 23-46.

(14) Freyer, M. W.; Lewis, E. A. Isothermal Titration Calorimetry: Experimental Design, Data Analysis, and Probing Macromolecule/Ligand Binding and Kinetic Interactions. In *Methods in Cell Biology*, Vol. 84; Academic Press, 2008; pp 79-113.
